# Mycobactin and clofazimine activity are negatively correlated in mycobacteria

**DOI:** 10.3389/fmicb.2025.1539139

**Published:** 2025-04-03

**Authors:** Martin I. Voskuil, Christopher R. Covey, Matthew J. Reichlen, Anushila Chatterjee, Breck A. Duerkop, Surendra Dawadi, Courtney C. Aldrich, Alexander Aaring

**Affiliations:** ^1^Department of Immunology and Microbiology, University of Colorado School of Medicine, Aurora, CO, United States; ^2^Department of Medicinal Chemistry, University of Minnesota, Minneapolis, MN, United States

**Keywords:** clofazimine, mycobactin, *Mycobacterium*, iron, siderophore

## Abstract

Clofazimine (CFZ) is an anti-leprosy drug shown to improve outcomes in treatment of multidrug-resistant tuberculosis (TB) and nontuberculous mycobacterial infections. Studies in *Mycobacterium tuberculosis* and *Mycobacterium avium* identified CFZ resistance mutations in the gene that encodes the MmpR5/MmpT5 regulator, which increase expression of the mycobactin (MBT) transporter, MmpS5/L5. We found exposure of *M. tuberculosis* to CFZ induced a pattern of gene expression that mirrored low iron conditions, including strong induction of genes that encode MBT synthesis and transport. We identified a corresponding increase in MBT levels indicating a role in iron homeostasis in CFZ activity. CFZ bactericidal activity against both *Mycobacterium smegmatis* and *M. tuberculosis* was increased in high iron conditions in which MTB synthesis and transport was limited. We show the presence of MBT correlated with decreased CFZ killing activity while inhibition of MBT synthesis increased killing. Considerable iron efflux was observed during CFZ treatment indicating iron loss may be a feature of CFZ anti-mycobacterial activity. CFZ solubility studies and CFZ-mediated reduction of free iron indicate a potential redox interaction between CFZ and iron. MBT or MBT flux across the cell envelope appears to block CFZ killing in *M. smegmatis* and potentially *M. tuberculosis*. The specific mechanism by which MBT inhibits CFZ lethality remains unclear but may involve, increased iron acquisition, the MmpS5/L5 MBT efflux pump, or the CFZ subcellular localization altered by the redox state and solubility of CFZ. CFZ has thus far been proven most effective against *Mycobacterium leprae*, which lacks MBT, indicating an understanding of the complex interaction of CFZ with iron acquisition systems may suggest more effective therapeutic applications.

## Introduction

According to the 2023 WHO Global Tuberculosis report, 176,000 or roughly 2% of the estimated 8.2 million new cases of *M. tuberculosis* infections were resistant to either rifampin or categorized as multidrug-resistant (MDR-TB) (WHO, 2023). Positive treatment outcomes in this group of MDR-TB cases stand at only 66% compared to the 88% success rate observed in drug-susceptible TB. A 2011–2014 study of MDR-TB patients in Bangladesh generated renewed interest in CFZ by demonstrating the utility of the drug in conjunction with gatifloxacin, ethambutol and pyrazinamide (Aung et al., 2014). This CFZ-containing regimen not only improved favorable outcomes to approximately 85%, comparable to treatment success of non-MDR-TB, but also allowed for decreased use of the highly toxic injectable kanamycin. A similar study in Brazil found that CFZ could effectively replace the more toxic prothioamide while improving patient outcomes (Van Deun et al., 2010). Yet another clinical study carried out in China between 2010 and 2013 found the addition of CFZ to individualized MDR-TB regimens increased cure rates from 54 to 74% (Tang et al., 2015). As of 2024, the WHO officially recommends CFZ as part of extended MDR-TB and extensively drug resistant-TB treatment regimens (WHO, 2022).

CFZ is a synthetic riminophenazine dye first synthesized in 1954 for use as an antimicrobial against *M. tuberculosis*. The drug exhibits strong *in vitro* activity against both *M. tuberculosis* and *Mycobacterium bovis*, indicating a potential antitubercular agent (Barry et al., 1957). However, CFZ was ineffective against *M. tuberculosis* in guinea pigs (Gopal et al., 2013) and nonhuman primates (Schmidt et al., 1955), complicating the clinical case for use of CFZ for TB treatment. CFZ was shown to be highly effective against *M. tuberculosis* in BALB/c mice but substantially less-so in C3HeB/FeJ mice if necrotic granuloma were permitted to form prior to drug treatment (Irwin et al., 2014), possibly explaining CFZ’s lack of efficacy in animal models that form necrotic granulomas. Regardless of its limitations, CFZ has proven valuable in treatment of multibacillary leprosy (Rees, 1969), while also showing utility as a component in the treatment of several clinically-relevant non-tuberculous mycobacteria (NTM) including *M. abscessus* and *M. avium* (Ruth et al., 2019; Lanoix et al., 2020; Ferro et al., 2015).

Widespread use of CFZ is complicated by a limited understanding of its overall killing mechanism. Early work identified the ability of CFZ to intercalate into DNA (Morrison and Marley, 1976) as well as disrupt cellular membranes as measured by the release of arachidonic acid from CFZ-treated bacteria (Anderson et al., 1988). More recent studies demonstrate unique roles for the cytochrome *c* and *bd* terminal oxidases (Lu et al., 2015) and show CFZ is reduced by the type-II NADH dehydrogenase (NDH-2) (Beites et al., 2019; Yano et al., 2011), indicating CFZ induces bacterial damage via the generation of reactive oxygen species. Additionally, a mechanism of CFZ resistance common to multiple mycobacterial species has been identified that involves the regulator of the MmpS5/L5 mycobactin (MBT) transport proteins, annotated MmpR5 in *M. tuberculosis* and MmpT5 in *M. intracellulare* (Milano et al., 2009; Radhakrishnan et al., 2014; Villellas et al., 2017), with a possible third example, MAB 2299c, from *M. abscessus* (Richard et al., 2019). Studies in *M. tuberculosis* and *M. intracellulare* established that mutations leading to higher expression of the *mmpS5/L5* genes resulted in increased CFZ resistance, pointing to a direct role for CFZ efflux via *mmpS5/L5* or potential role for siderophore transport in resistance (Ismail et al., 2019b; Hartkoorn et al., 2014; Alexander et al., 2017). Interestingly, these mutations in all three species also confer resistance to bedaquiline, with multiple studies demonstrating 4-fold or more increases in MIC in *mmpR5* mutants of *M. tuberculosis* (Villellas et al., 2017; Ismail et al., 2019a; Hartkoorn et al., 2014).

*M. tuberculosis* uses MBT/carboxymycobactin (cMBT) for the acquisition of ferric (Fe^3+^) iron from biological repositories such as transferrin and lactoferrin (Sritharan, 2016). The iron acquisition system employs the polar cMBT variant with a shortened alkyl tail that is secreted into the aqueous extracellular environment where it abstracts Fe^3+^ from host proteins. cMBT then delivers Fe^3+^ to MBT found in the mycobacterial cellular envelope where it is internalized and reduced as ferrous (Fe^2+^) iron. Some mycobacteria, e.g., *M. smegmatis*, also produce exochelin, an additional soluble siderophore (Sharman et al., 1995). Exochelin can almost entirely supplant the function of cMBT in *M. smegmatis*, likely due to the overlapping extracellular siderophore roles of cMBT and exochelin in acquiring Fe^3+^ (Ratledge and Ewing, 1996). Synthesis of MBT and cMBT involves the generation of a common core peptidic scaffold containing three bidendate chelating moieties capable of binding Fe^3+^. In addition to the proteins necessary for producing MBT and cMBT, other systems are involved in both the mobilization of these two siderophores across the bacterial envelope and for the internalization of the acquired iron. As well as the aforementioned MmpS5/L5 system, proteins necessary for siderophore transport include the related MmpS4/L4 and the ESX-3 systems, while internalization is facilitated by IrtA/B (Rodriguez and Smith, 2006; Wells et al., 2013; Siegrist et al., 2009). Intracellular accumulation of MBT and cMBT in the absence of MmpS4/S5 siderophore transporters results in a toxic self-poisoning of *M. tuberculosis* (Wells et al., 2013; Jones et al., 2014). Loss of the MmpS5-MmpL5 transporter also leads to increased sensitivity to CFZ, bedaquiline, and rifabutin indicating efflux of large nonlinear hydrophobic compounds with aromatic rings (Meikle et al., 2023). *M. tuberculosis* can also acquire iron via a heme uptake system (Jones and Niederweis, 2011). The heme uptake system utilizes proteins which are largely distinct from those necessary for Fe^3+^ uptake, although components of the ESX-3 system and the MmpS4 protein appear to be necessary for both heme and Fe^3+^ acquisition (Zhang et al., 2020).

In this study, we sought to investigate the relationship between iron homeostasis and CFZ killing with an emphasis on MBT to better understand the interplay between iron availability and transport and mechanisms that influence CFZ activity. We found that CFZ induced an *M. tuberculosis* low iron transcriptional response, including induction of MBT synthesis and transport genes, and conditions that necessitated MBT utilization had a strong negative impact on CFZ killing.

## Materials and methods

### Bacterial strains and culture methods

*M. smegmatis* mc^2^155 was used both as the wild-type and parental strain for siderophore mutants. ∆*mbtD* and ∆*fxbA* strains were generated using the p0004S allelic-exchange system, as described (Larsen et al., 2007). Experiments with virulent *M. tuberculosis* were performed using strain Erdman TMCC 107 gifted by Dr. Anne Lenaerts (Colorado State University).

*M. smegmatis* and *M. tuberculosis* were grown in Dubos media consisting of 2 g asparagine, 0.5 g casitone, 1 g KH_2_PO_4_, 2.5 g Na_2_HPO_4_, 10 mg MgSO_4_·6H_2_O, 0.5 mg CaCl_2_, 0.1 mg ZnSO_4_, 0.1 mg CuSO_4_, 7.5 g glucose, 5 g bovine serum albumin fraction 5, 1.7 g NaCl and 2.5 mL 20% Tween-80 in 1 liter of diH_2_O unless otherwise stated; media was adjusted to pH 6.64 with HCl. Standard Dubos medium was supplemented with 50 mg/L ferric ammonium citrate equating to a total iron content of approximately 190 μM. In the absence of this supplement, residual iron levels were approximately 2 μM, as determined using a bathophenanthraline disulfonate iron detection assay (Nilsson et al., 2002). Avirulent *M. tuberculosis* ML1600 (∆*mbtD, ∆RD1* ∆*panCD*), gifted by Dr. Michael Neiderweis (University of Alabama, Birmingham), was grown in Middlebrook 7H9 prepared from powdered base (Sigma Aldrich) with 0.2% casitone, 24 μg/mL pantothenic acid, 20 μM porcine hemin and 0.05% Tween-80, as previously described (Wells et al., 2013). All bacterial cultures were grown at 37°C with stirring in 125 mL Erlenmeyer flasks prior to experiments. All chemicals were obtained from Sigma Aldrich unless otherwise specified.

### CFZ exposure assays

*M. smegmatis* was diluted from freezer stocks to OD_600_ 0.0006 and grown for 19 h in Dubos medium. Cultures were then diluted to OD_600_ 0.03, dispensed in 1.5 mL aliquots into 14 mL snap cap tubes which were loosely capped and incubated for 90 min at 37°C with shaking at a 30° angle. Drugs were added to the tubes at indicated concentrations and the tubes were returned to the incubator. Tubes were assayed for CFU at indicated timepoints via plating in triplicate on Dubos media plates containing 4 g/L activated charcoal. *M. smegmatis* experiments were conducted over 6 days to observe the full extent of CFZ lethal activity.

*M. tuberculosis* Erdman was settled at 37°C for 4 days and avirulent ∆*mbtD M. tuberculosis* strain ML1600 for 6 days at 37°C in 10 mL of media before being brought to 50 mL. Strains were incubated with stirring at 37°C for 6 days with periodic monitoring and dilution to keep cultures between OD_600_ 0.05 and 0.5. For killing assays, cultures were diluted to OD_600_ 0.05 and dispensed in 2 mL aliquots to 24 mL borosilicate tubes containing sterile magnetic stir bars; ML1600 cultures were diluted to OD_600_ 0.1. Erdman cultures were then incubated with stirring at 37°C with 5% CO_2_ for 150 min and without CO_2_ for ML1600. Drug or vehicle was introduced to tubes as indicated and tubes were returned to incubator. Cultures were assayed for CFU at indicated timepoints via plating.

Filter-sterilized aliquots of the antibiotics CFZ and kanamycin were stored at -80°C and norfloxacin at 4°C before use. Chlorpromazine was prepared fresh prior to each use. Cell-associated mycobactin J (MBTJ) isolated from *M. avium* subsp. *Paratuberculosis* (Schwartz and De Voss, 2001) and obtained from Allied Monitor was dried down in 200 μg aliquots and resuspended in ethanol prior to use. Salicyl-AMS was synthesized as previously described (Somu et al., 2006).

### Mycobactin labeling and quantification

*M. smegmatis* and *M. tuberculosis* were cultured with or without CFZ in the indicated medium under the aforementioned growth conditions for 24 h with 0.1 μCi/mL 7–^14^C salicylic acid (American Radiolabeled Chemicals). Cell pellets were extracted for cell-associated MBT, as previously described (Wells et al., 2013). Cell pellets were boiled in the presence of 4% SDS for 2 h then quantified using the BCA protein assay. Extracts were normalized to 100 mg total protein for *M. smegmatis*, 50 mg for *M. tuberculosis*. Samples were separated via thin-layer chromatography on 0.25 mm glass-backed silica plates using 2:3:3 petroleum ether:*n*-butanol:ethyl acetate solvent system (De Voss et al., 2000) which were then exposed to a Kodak Imaging screen (K-screen). Radiation was visualized and quantified using a Typhoon FLA 7000 molecular imager.

### CFZ solubility assay

CFZ at a concentration of 100 μM was added to an aqueous buffer solution consisting of 1 g KH_2_PO_4_ and 2.5 g Na_2_HPO_4_ in 1 liter of water, pH adjusted to 6.64. Increasing concentrations of ferric ammonium citrate were introduced, with or without the addition of 50 mM sodium ascorbate in 14 mL snap cap tubes. Samples were incubated with shaking for 30 min after which samples were centrifuged, the buffer was decanted, and the tube washed with 1 mL DMSO to remove precipitate. The wash was read at OD_454_ and compared to a standard curve consisting of known quantities of CFZ in DMSO.

### Fe^2+^ accumulation assay

*M. tuberculosis* H37Rv was grown in the Rapid Anaerobic Dormancy (RAD) model as previously described (Leistikow et al., 2010) using DTA broth supplemented with either 50 or 250 mg/L of ferric ammonium citrate, equating to 190 μM and 950 μM total iron, respectively. Cultures were grown in the RAD model for 8 days to achieve complete O_2_ depletion. Cultures were then transferred to an anaerobic environmental chamber (Shell lab, Cornelius, OR, USA), unsealed and challenged with either 106 μM CFZ or a DMSO vehicle control. Cultures were resealed, removed from the anaerobic chamber and incubated at 37°C. Cultures were assayed for the accumulation of Fe^2+^ via a colorimetric assay using the bidentate chelating agent ferene (3-(2-pyridyl)-5,6-bis(2-(5-furyl sulfonic acid))-1,2,4-triazine) which forms a stable complex with Fe^2+^ with a peak absorbance at 593 nm (Eskelinen et al., 1983). Fe^2+^ accumulation was assayed at 5, 24, 48, 120, and 192 h following the addition of CFZ. At indicated timepoints, cultures were transferred to the anaerobic chamber. Cultures were unsealed, 1.0 mL aliquots were collected and centrifuged for 5 min at 3,000 rpm to pellet out cell material. Supernatant was removed and supplemented with ferene at a final concentration of 50 μM. Samples were incubated for 5 min at room temperature to allow for complete color development. Samples were removed from the anaerobic chamber and assayed for Fe^2+^ by measuring the absorbance at 593 nm. Total sample Fe^2+^ was inferred by plotting against a standard curve prepared using aqueous solutions of FeSO_4_.

### Expression profiling

RNA was isolated from *M. tuberculosis* as previously described (Rodriguez et al., 2002) from cultures challenged with either CFZ or DMSO. RNA was sequenced using an Illumina NovaSeq6000 platform with a minimum of 60 million reads per sample. Sequencing reads were mapped to *M. tuberculosis* Erdman (NC_AP012340) using CLC genomics workbench. To normalize raw gene count by transcript length and sequence depth, the gene expression values were calculated as reads per kilobase per million. Differential transcript expression between the control and experimental samples was calculated using generalized linear model to correct for library size variations between samples.

### ^55^Fe efflux

*M. smegmatis* cultures were pre-grown for 19 h in 2 μM iron Dubos medium supplemented with 1 μCi/mL ^55^FeCl_3_ (American Radiolabeled Chemicals). Bacilli were washed before outgrowth, resuspended in either 2 μM or 190 μM iron Dubos medium and treated with CFZ or DMSO as indicated by the assay. Samples were harvested at indicated timepoints with 1 mL per sample spun down for 3 min twice. 500 μL of supernatant was analyzed using a Beckman LS 6500 liquid scintillation counter on the ^3^H channel for 8 min.

### RNAseq accession numbers

Expression profiling data from this study are available at the ArrayExpress at EMBL-EBI^1^ under accession number E-MTAB-9350.

## Results

### CFZ exposure induced iron acquisition response

**Figure 1:**
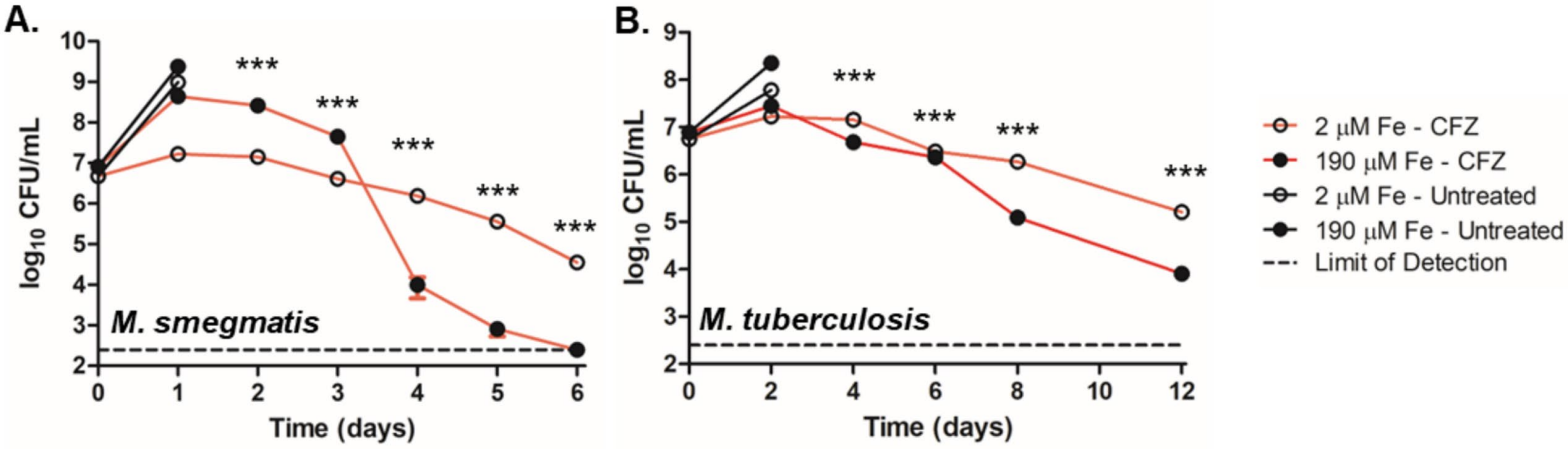
Low iron conditions reduced CFZ killing. **(A)** *M. smegmatis* and **(B)** *M. tuberculosis* were challenged with 50 and 20 μM CFZ, respectively, in medium containing either 190 μM (closed circles) or 2 μM iron (open circles). CFU were enumerated by plating on charcoal-containing solid medium at indicated timepoints and analyzed using an unpaired student’s t-test. Panel A represents the average of nine experimental replicates while panel B represents eight experimental replicates. Data represent the mean log_10_ CFU counts ± SEM of a with a 250 CFU limit of detection and were analyzed using an unpaired student’s t-test. ***, *p* < 0.001.

To determine how *M. tuberculosis* adapts to CFZ exposure we performed RNAseq transcriptional profiling. Cultures were subjected to either 20 μM CFZ or DMSO vehicle control for 24 h before being harvested for RNA isolation and RNAseq analysis. Exposure of *M. tuberculosis* to 20 μM CFZ resulted in several days of growth before the onset of killing to the limit of detection by 6 days (Figure 1B). Alignment of reads to a reference genome (Miyoshi-Akiyama et al., 2012) demonstrated a significant (*p* < 0.05) increase of 2-fold or more in 353 genes (Supplementary Table S1) and a 2-fold or more decrease in 178 genes (Supplementary Table S2). A prominent change observed during CFZ treatment was the substantially increased expression of genes in the IdeR regulon involved in iron homeostasis (Table 1). IdeR differentially regulates iron-associated genes based on the binding of intracellular Fe^2+^ and ensures that iron storage proteins are expressed when iron is replete and the systems necessary for *de novo* siderophore production and iron acquisition are expressed when iron is scarce (Gold et al., 2001; Rodriguez et al., 2002).

**Table 1:** CFZ exposure led to expression changes of IdeR-regulated genes consistent with low iron conditions.^a^

Rv No.^b^	Gene	Fold change (CFZ vs. DMSO)	Function
Repressed by IdeR			
0116c	*ldtA*	1.48	Peptidoglycan biosynthesis
0282	*eccA3*	3.92	ESX-3 type-VII secretion
0283	*eccB3*	3.75	
0284	*eccC3*	3.31	
0285	PE5	3.94	
0286	PPE4	3.15	
0287	*esxG*	4.06	
0288	*esxH*	2.71	
0289	*esxG3*	3.07	
0290	*eccD3*	2.97	
0291	*mycP3*	3.19	
0292	*eccE3*	2.18	
0450c	*mmpL4*	5.23	MBT transport
0451c	*mmpS4*	7.99	
0587	*yrbE2A*	−1.02	Conserved hypothetical protein
0766c	*cyp123*	−1.24	Probable cytochrome p450
1343c	*lprD*	22.0	MBT biosynthesis
1344	*mbtL*	38.4	
1345	*mbtM*	20.2	
1346	*mbtN*	30.0	
1347c	*mbtK*	11.6	
1348	*irtA*	11.8	MBT ABC transport
1349	*irtB*	10.1	
1519		9.86	Conserved hypothetical protein
2122c	*hisE*	6.37	Histidine biosynthesis
2123	PPE37	58.2	PPE family protein
2377c	*mbtH*	23.0	MBT biosynthesis
2378c	*mbtG*	21.4	
2379c	*mbtF*	15.5	
2380c	*mbtE*	25.9	
2381c	*mbtD*	34.0	
2382c	*mbtC*	59.2	
2383c	*mbtB*	43.9	
2384	*mbtA*	13.2	
2385	*mbtJ*	19.3	
2386c	*mbtI*	41.9	
3402c		37.9	Conserved hypothetical protein
3403c		12.8	Hypothetical protein
3839	*fdhD*	88.4	Formate dehydrogenase
3840	*fdhF*	10.7	
Induced by IdeR			
0009	*ppiA*	−2.38	Probable isomerase
0338c		−4.71	Probableiron-sulfur-binding reductase
1552	*bfrA*	−3.53	Bacterioferritin iron storage
3841	*bfrB*	−4.78	

a IdeR-regulated genes identified previously (Rodriguez et al., 2002).

b Erdman notation from sequence assembly converted to H37Rv notation due to more widespread adoption of the latter.

The two MBT biosynthetic operons comprising *mbtA-mbtK* and *mbtL-mbtN* were induced from 11- to 59-fold during CFZ exposure (Table 1), as were *irtA* and *irtB* associated with internalizing MBT-bound iron (Rodriguez and Smith, 2006). Components of the MmpS4/L4 and ESX-3 MBT transport systems (Wells et al., 2013; Siegrist et al., 2009), which fall under IdeR control, were also upregulated, albeit by a lesser degree than observed for the *mbt* and *irt* operons, with induction ranging from approximately 2- to 8-fold over DMSO vehicle control. The iron storage bacterioferritin genes, *bfrA* and *bfrB*, are induced by IdeR under high iron conditions and were downregulated by 3.5- and 4.8-fold by CFZ, respectively.

**Figure 2:**
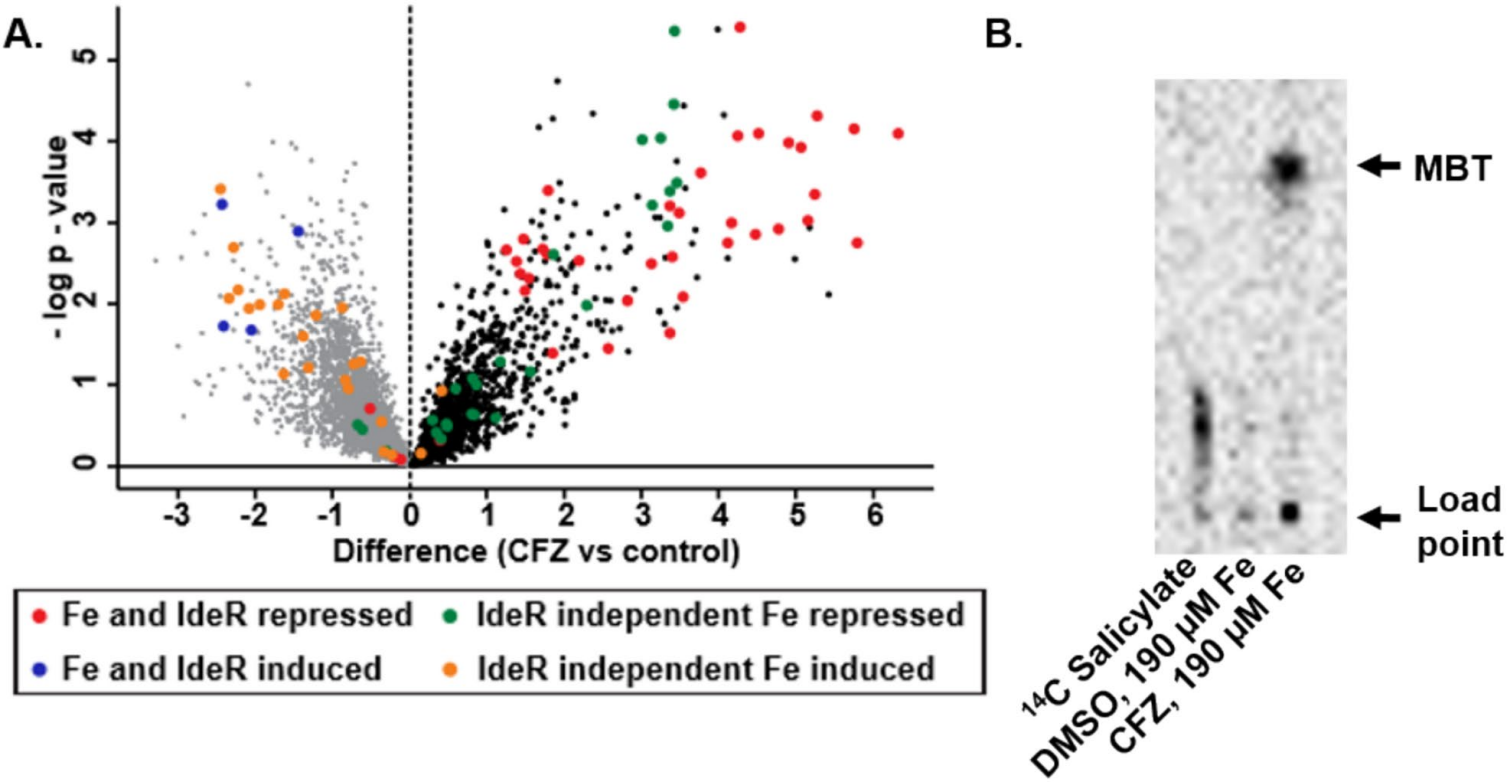
CFZ exposure induced *M. tuberculosis* iron-starved expression profile. **(A)** *M. tuberculosis* genes previously established as differentially regulated by iron availability were presented in a volcano plot with groupings determined by whether high iron repressed or induced gene expression and whether the effect was mediated by IdeR (Rodriguez et al., 2002). **(B)** MBT production was verified using ^14^C salicylate-labeling of MBT. Presumptive MBT spots were consistent with R_f_ values established for MBT in selected solvent system (42).

The impact of CFZ on the IdeR regulon and other iron-regulated genes, as defined by Rodriguez et al. (2002), is represented in a volcano plot with genes grouped by both their dependence on IdeR and whether iron increases or decreases expression (Figure 2A). Expression analysis revealed a pattern of iron-associated gene expression consistent with a low iron response for both IdeR-dependent and independent genes. The production of MBT during conditions used for expression profiling was demonstrated using ^14^C-salicylate MBT labeling (Figure 2B). The DMSO vehicle control culture produced no observable MBT as would be expected for iron replete conditions, but CFZ exposure resulted in a visible accumulation of cell-associated MBT (De Voss et al., 2000), validating the CFZ-associated increase in MBT-associated gene expression.

### CFZ lethality was diminished in low iron conditions

We next investigated the role of environmental iron availability on CFZ killing. *M. smegmatis* and *M. tuberculosis* were grown with either the standard 50 mg/L ferric ammonium citrate supplement (190 μM total iron) or 0 ferric ammonium citrate (2 μM total iron) referred to as high and low iron, respectively. High levels of MBT were observed from cell extracts of *M. smegmatis* grown under low iron but absent from cultures grown at high iron, confirming iron limiting and replete conditions (Supplementary Figure S1). *M. smegmatis* cultures were treated with 50 μM CFZ and *M. tuberculosis* cultures with 20 μM CFZ. Results indicated a stark impact of low iron on CFZ killing in *M. smegmatis* (Figure 1A). High iron cultures exhibited a one-day period of growth of 1.7-log_10_ that transitioned to a rapid killing phase after 3 days and terminated at the limit of detection (250 CFU mL^−1^) after 6 days, constituting a 6.0-log_10_ reduction in CFU from the 2-day high. Meanwhile low iron cultures experienced a relatively slow decline in CFU, dropping only 2.7-log_10_ over the same period. The differential killing by CFZ of *M. tuberculosis*, at high and low iron was less pronounced than in *M. smegmatis* (Figure 1B), but a significant difference was observed.

### Presence of MBT correlated with reduced CFZ lethality

**Figure 3:**
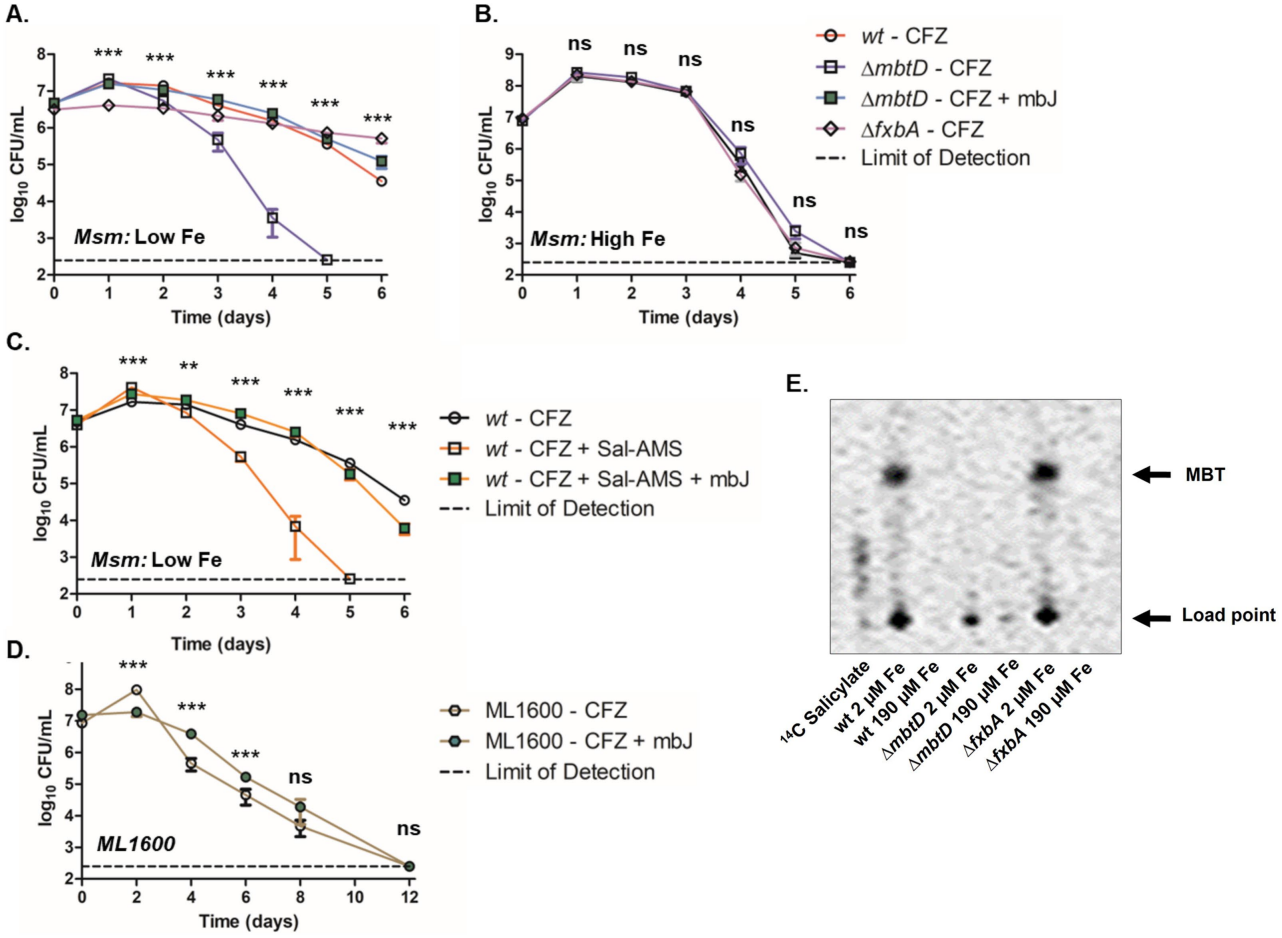
Absence of MBT correlated with increased CFZ killing. *M. smegmatis* wild-type (circles), ∆*mbtD* (squares) or ∆*fxbA* (diamonds) background were grown and challenged with 50 μM CFZ in either low, 2 μM iron **(A)** or high, 190 μM iron **(B)** iron medium. *M. smegmatis* ∆*mbtD* in low iron was also challenged with CFZ in the presence of 5 μM MBTJ (green squares). **(C)** Wild-type *M. smegmatis* was also challenged with CFZ alone (open circles), CFZ + 16 μM Salicyl-AMS (open squares) or CFZ + 16 μM Salicyl-AMS + 5 μM MBTJ (filled squares) in medium with low iron. **(D)** ∆*mbtD* avirulent *M. tuberculosis* cultures (ML1600) was challenged with 20 μM CFZ (open hexagons) or with CFZ and 5 μM MBTJ supplement (filled hexagons). **(E)** Production of MBT was visualized using ^14^C-salicylate, after 24 h of CFZ exposure at conditions plated in Panels **(A,B)**. Cultures from panels **(A–D)** were plated in triplicate for CFU at indicated timepoints. Analysis for panels **(A–C)** was performed using the Kruskal-Wallis test and an unpaired student’s t-test was performed for panel **(D)**. Data represent the mean log 10 CFU counts of 9 (panels **A–C**) or 7 (panel **D**) experimental replicates with a 250 CFU limit of detection. **p < 0.01; ***p < 0.001.

To determine why iron limitation reduced CFZ killing, we assessed the role of individual siderophores. *M. smegmatis* mutants were generated to specifically deprive the bacteria of each class of siderophore: deletion of the polyketide synthase-encoding *mbtD* prevents production of both MBT and cMBT while deletion of the putative formyltransferase-encoding *fxbA* prevents the production of exochelin (Zhu et al., 1998; Fiss et al., 1994; Quadri et al., 1998; McMahon et al., 2012). Deletion of *mbtA* or *mbtB* would have been sufficient to block MBT and cMBT production (Lun et al., 2013; De Voss et al., 2000); however, ∆*mbtD* was chosen in order to phenocopy the MBT mutant in avirulent *M. tuberculosis* ML1600, used in this study (Jones et al., 2014). Mycobactin and exochelin minus strains as well as wild-type *M. smegmatis* were then challenged with 50 μM CFZ at low iron (Figure 3A). Inhibition of MBT production led to increased killing at day 2 and no detectable viable *M. smegmatis* after 5 days exposure. Inhibition of exochelin production did not increase killing but resulted in a more static effect of CFZ relative to that observed in the wild-type. The increased killing of ∆*mbtD* cultures by CFZ was complemented by the addition of 5 μM exogenous MBTJ, serving to confirm the impact of MBT on CFZ activity. The impact of MBT on *M. smegmatis* CFZ killing was further interrogated with the MbtA inhibitor Salicyl-AMS (Lun et al., 2013). Exposure of wild-type *M. smegmatis* to both CFZ and 16 μM Salicyl-AMS increased killing that was comparable to the impact of the *mbtD* deletion (Figures 3A,C). Addition of 5 μM MBTJ to the ∆*mbtD* strain prevented CFZ killing as it did when added to the Salicyl-AMS treated culture. Wild-type ∆*mbtD* and ∆*fxbA M. smegmatis* strains displayed no difference in killing by CFZ under high iron conditions (Figure 3B). Use of ^14^C salicylate to measure MBT production indicated that MBT was present in conditions with low CFZ activity and absent from conditions that exhibited high activity (Figure 3E). Interestingly, wild-type *M. smegmatis* produced MBT during CFZ exposure but less than low iron cultures alone (Supplementary Figure S1). Therefore, CFZ did not strongly induce *M. smegmatis* MBT synthesis as it did in *M. tuberculosis* (Figure 2B), likely due to the ability to use exochelin in *M. smegmatis*.

With regards to *M. tuberculosis*, CFZ challenge of ML1600 ∆*mbtD* demonstrated a statistically significant difference for the first 6 days between cultures treated with CFZ or with CFZ plus 5 μM MBTJ. However, the survival advantage observed in MBTJ supplemented cultures was not significant by day 8 and all culture viability fell below the limit of detection by day 12 (Figure 3D).

**Figure 4:**
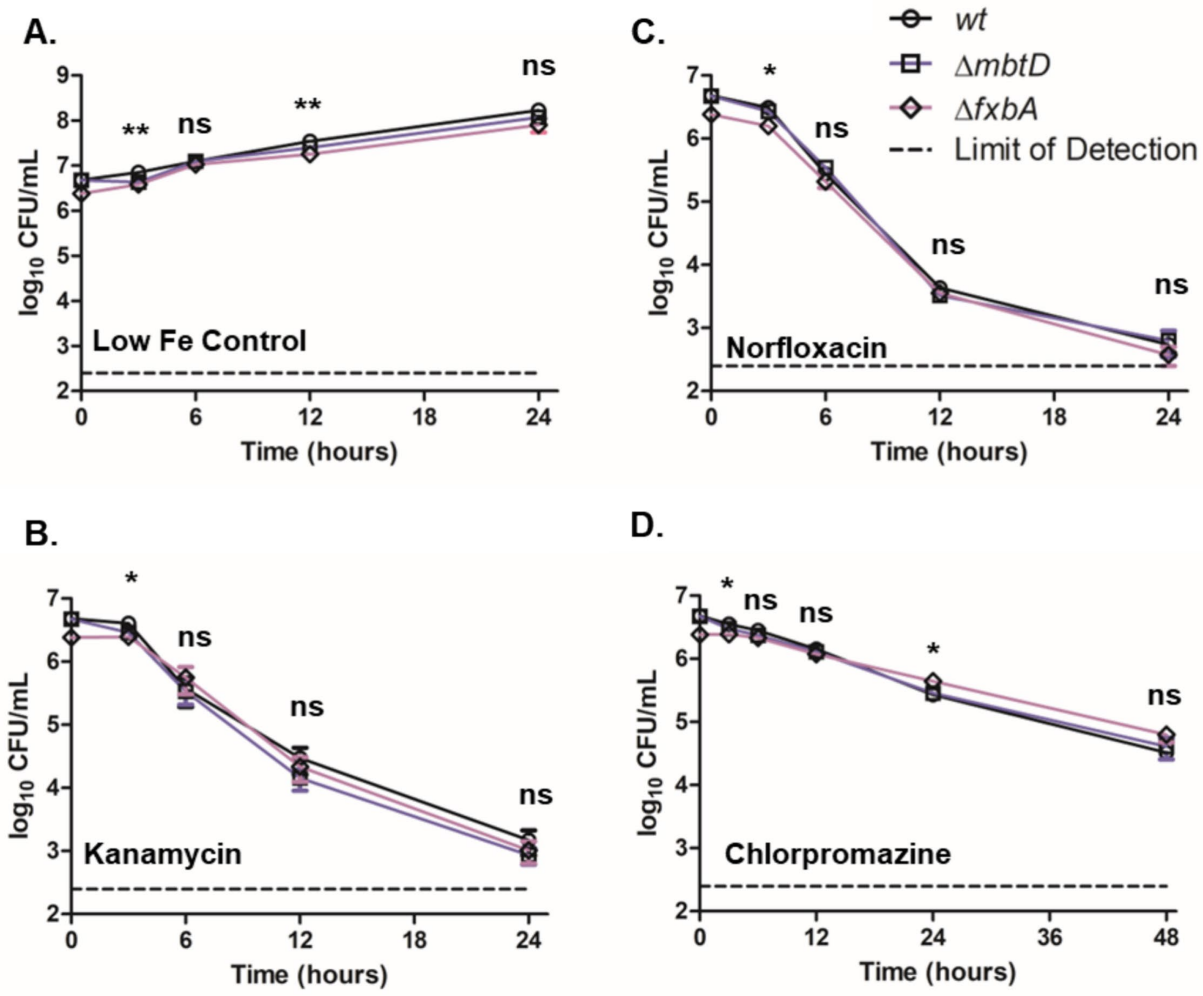
Presence of siderophores did not substantially impact *M. smegmatis* low iron growth or killing by kanamycin, norfloxacin or chlorpromazine. **(A)** *M. smegmatis* siderophore mutants were monitored for growth in low iron medium as well as for killing by **(B)** 300 nM kanamycin, **(C)** 25 μM norfloxacin or **(D)** 200 μM chlorpromazine. Wild-type (circles), ∆*mbtD* (squares), and ∆*fxbA* (diamonds) *M. smegmatis* were plated for CFU onto charcoal-containing solid medium at indicated timepoints and analyzed using Kruskal-Wallis test. Data represent the mean log_10_ CFU counts ± SEM of 6 experimental replicates with a 250 CFU limit of detection. *p < 0.05; **p < 0.01.

We next investigated the response of the *M. smegmatis* siderophore mutants to other antimicrobials to determine if the effect of MBT on CFZ activity was specific to CFZ. Wild-type, ∆*mbtD*, and ∆*fxbA* cultures were grown with low iron and monitored for CFU during challenge with 25 μM norfloxacin, 300 nM kanamycin, or 200 μM chlorpromazine (Figures 4A–D). Statistically significant differences were observed at 3 and 12 h in control cultures without antibiotic but 24-h growth indicated that strains were within one doubling of each another. Killing by norfloxacin, kanamycin, or chlorpromazine was remarkably similar between strains. Slight statistical significance was observed at 3 h for all drugs and additionally at 24 h for chlorpromazine, but these differences did not alter the overall trends observed for each agent.

### MBTJ did not prevent CFZ killing of *M. smegmatis* at high iron or when added late during CFZ exposure

**Figure 5:**
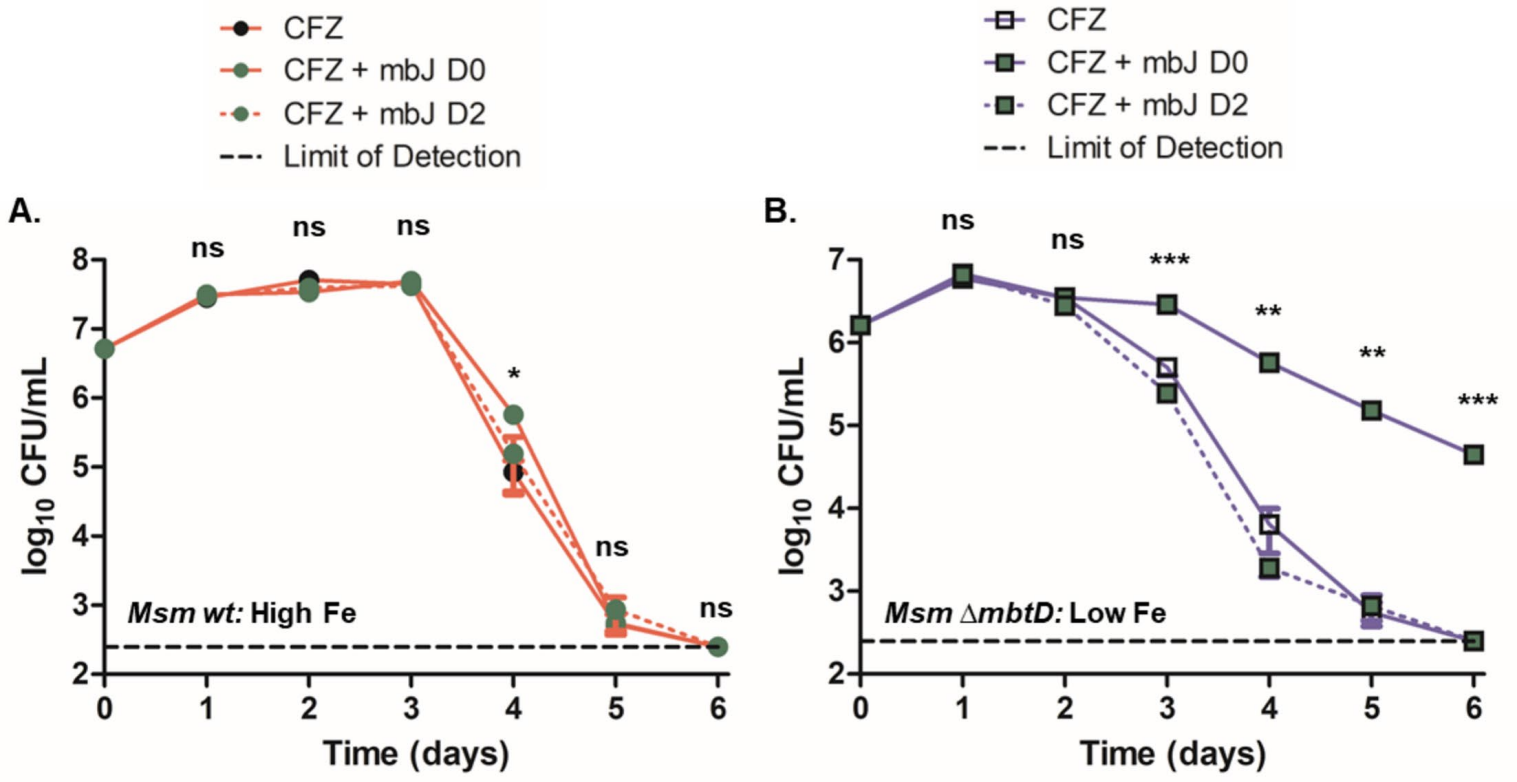
Presence of MBT was not sufficient to prevent CFZ killing under all conditions. *M. smegmatis* **(A)** wild-type and **(B)** ∆*mbtD* were treated with 50 μM CFZ in **(A)** high iron or **(B)** low iron medium either alone or in conjunction with 5 μM MBTJ added concurrently or 2 days post-CFZ addition. Cultures were plated for CFU at indicated timepoints and analyzed using Kruskal-Wallis test. Data represent the mean log_10_ CFU counts ± SEM of six experimental replicates with a 250 CFU limit of detection. *p < 0.05; **p < 0.01; ***p < 0.001.

Wild-type *M. smegmatis* cultures grown in high iron and challenged with CFZ did not demonstrate substantially reduced killing when 5 μM MBTJ was added concurrently with CFZ or 2 days post CFZ addition (Figure 5A). Only the day 4 timepoint showed a significant difference between the three conditions; however, all three cultures reached the limit of detection by day 6, indicating that the difference observed was minimal. ∆*mbtD* cultures grown in low iron displayed rescue by MBTJ only when added concurrently with drug; MBTJ addition after 2 days of CFZ exposure even increased killing over CFZ alone by approximately two-fold at days 3 and 4 (Figure 5B). Therefore, MBTJ addition did not rescue CFZ killing in high iron conditions, nor did it complement the ∆*mbtD* cultures when given 2 days after CFZ exposure began. As such, the presence of MBT alone was not sufficient to prevent CFZ killing.

### CFZ-induced iron efflux did not correlate with bactericidal activity

**Figure 6:**
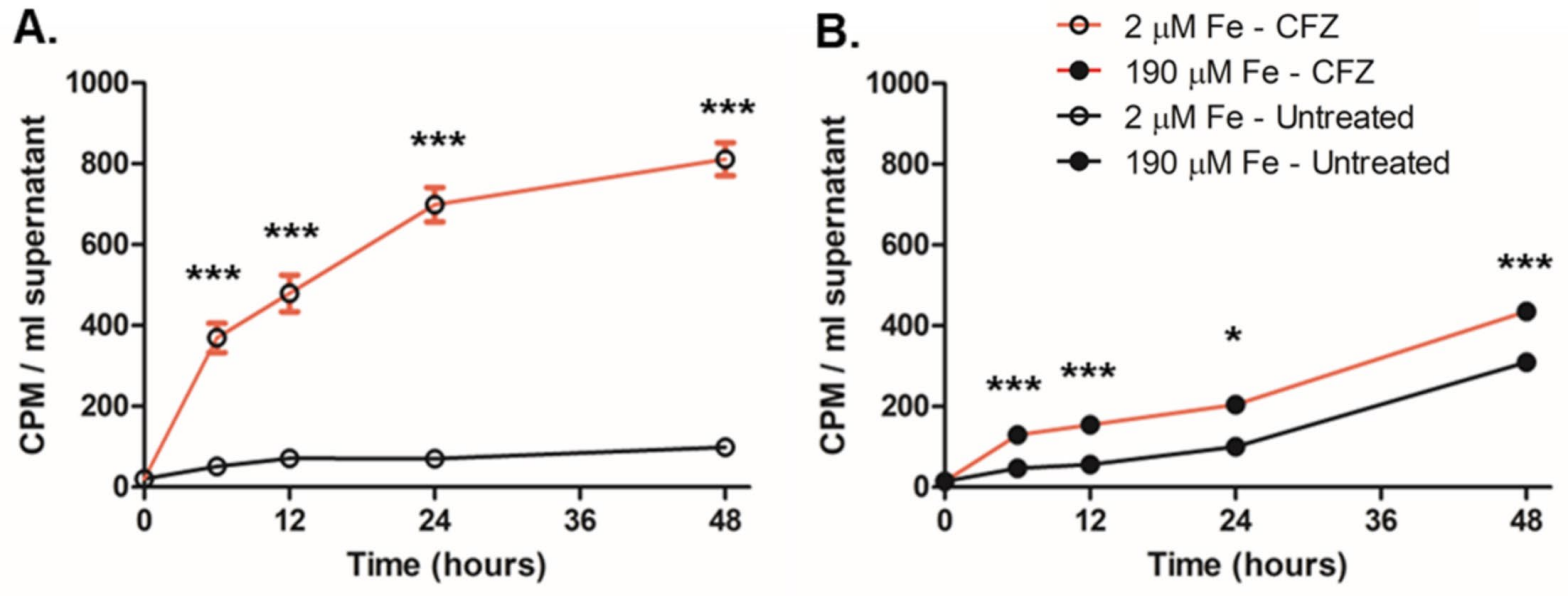
CFZ promoted iron efflux from *M. smegmatis*. *M. smegmatis* pre-grown in medium supplemented with 1 μCi/mL ^55^FeCl_3_ was washed and transferred into either **(A)** low or **(B)** high iron medium and treated with either 50 μM CFZ (red line) or DMSO vehicle control (black line). At indicated timepoints, cultures were pelleted and supernatant was read for ^55^Fe. Data represent the mean CPM/ml ± SEM of 6 experimental replicates performed in duplicate. Panels **(A,B)** were analyzed using an unpaired student’s t-test. *p < 0.05; ***p < 0.001.

We next sought to investigate the broader impact of CFZ on intracellular iron levels given the importance of MBT in CFZ killing. Intracellular iron efflux was detected and quantified by preloading *M. smegmatis* with ^55^FeCl_3_ and challenging with CFZ. Substantial iron loss in the presence of CFZ was observed within 6 h in cultures grown in low iron medium that would be expressing MBT (Figure 6A) while high iron cultures lost iron at a far slower rate (Figure 6B). Low iron loss occurred even though high iron conditions resulted in far greater CFZ killing than low iron conditions. Plating controls confirmed CFZ did not result in death during the timepoints tested, making the presence of supernatant ^55^Fe from lysis unlikely (Supplementary Figure S2).

### Iron and a reducing environment allowed for aqueous solubility of CFZ

**Figure 7:**
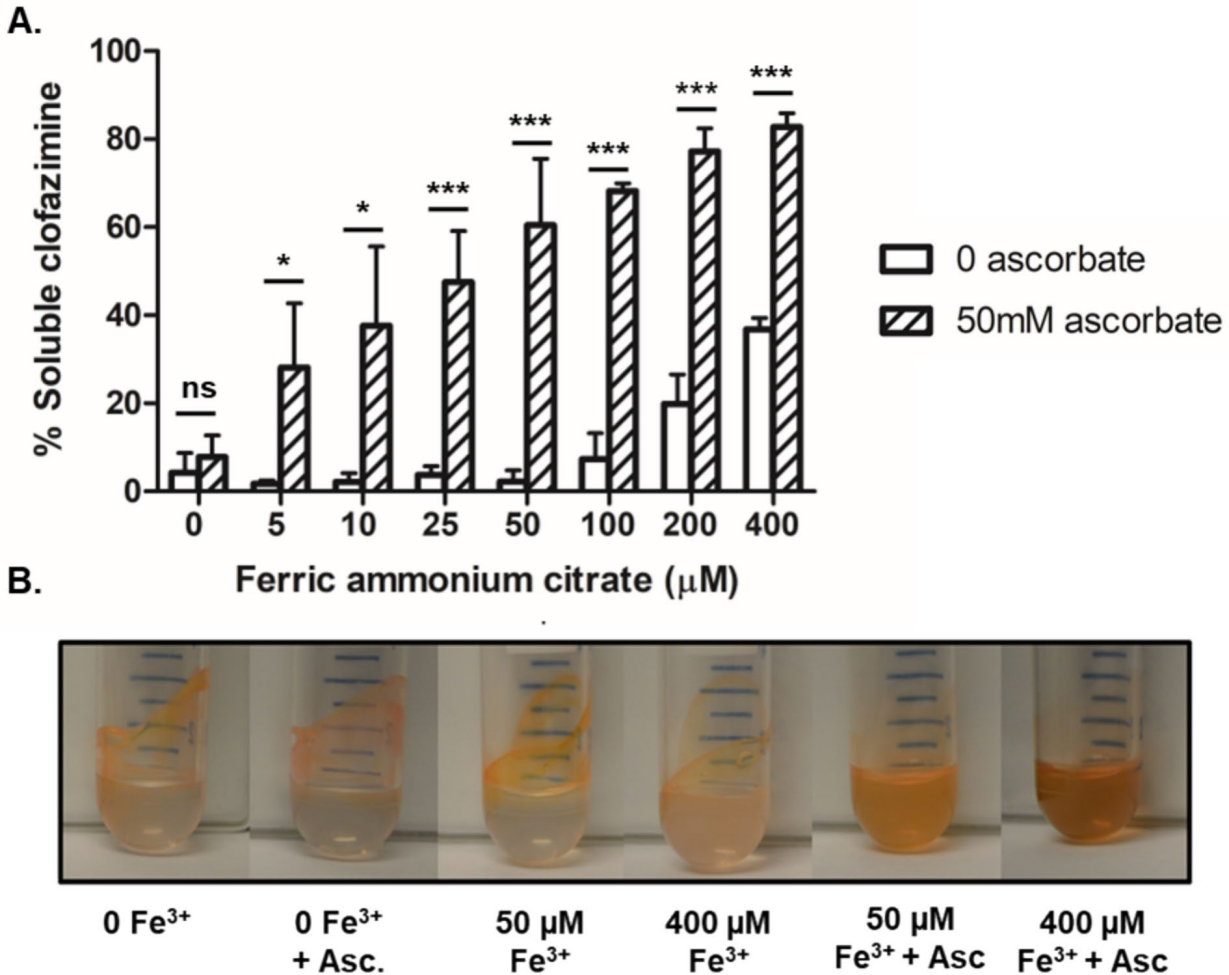
Increasing Fe^2+^ allowed CFZ to remain soluble. **(A)** The ability of CFZ to remain in aqueous buffer solution in the presence of increasing iron, both with the reducing agent sodium ascorbate (diagnol bars) or without (white bars), was quantified and **(B)** photographed. Data for panel **(A)** represents the mean ± SEM of four experimental replicates. Comparison between four experimental replicates for panel **(A)** was performed using unpaired student’s t-test. *p > 0.05; ***p > 0.001.

It has been demonstrated via fluorescence emission spectroscopy that CFZ is capable of interacting with a number of divalent transition metal cations including biologically relevant elements such as Cu^2+^, Zn^2+^, and Mn^2+^ (Mohd et al., 2011). In aerobic conditions, extracellular iron exists primarily in the oxidized Fe^3+^ form. We investigated the impact of increasing Fe^3+^ on CFZ solubility in buffer both alone and with addition of 50 mM sodium ascorbate as a reducing agent (Figures 7A,B). The reducing agent alone did not significantly improve CFZ solubility. Buffer alone and buffer plus sodium ascorbate each enabled less than 10% of CFZ to be maintained in solution after 30 min. However, increasing amounts of iron in the presence of sodium ascorbate led to improved solubility, while Fe^3+^ alone did not improve solubility over buffer alone until at least 100 μM was added. The combination of iron and a reducing environment shifted CFZ from less than 10 to 82% soluble in aqueous solution as a function of iron concentration indicating an interaction of Fe^2+^ with CFZ or reduced CFZ with Fe^3+^.

### Anaerobic survival during CFZ exposure increased with iron availability and correlated with the accumulation of extracellular Fe^2+^

**Figure 8:**
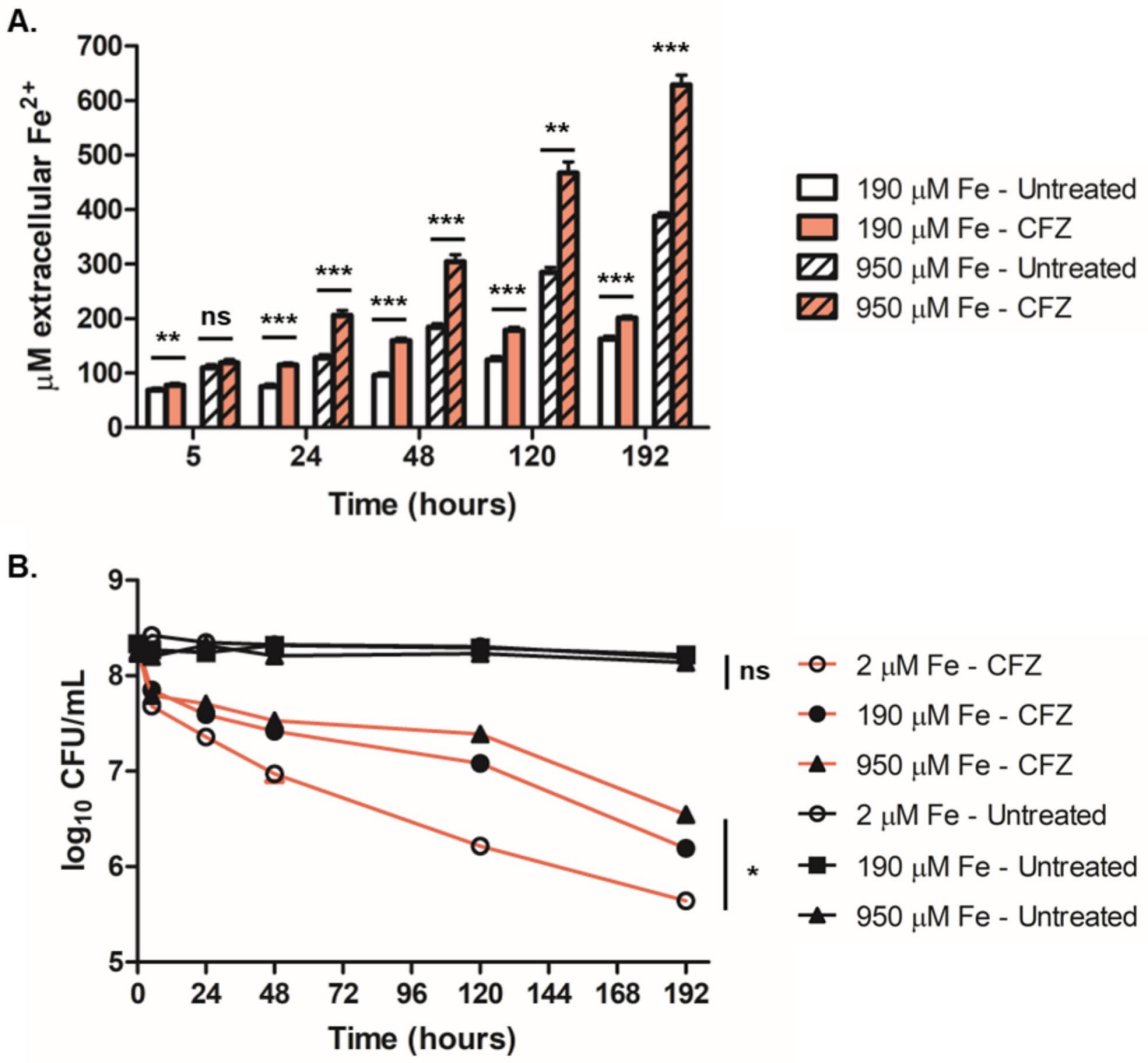
Survival during anaerobic CFZ exposure increased with iron availability and correlated with accumulation of extracellular Fe^2+^. **(A)** Extracellular Fe^2+^ was assayed at 190 μM (open bars) and 950 μM (lined bars) both with CFZ (red) and DMSO (white) at indicated timepoints using the colormetric Fe^2+^ chelator, ferene. **(B)** *M. tuberculosis* cultured in an anaerobic model for 8 days prior to CFZ addition were treated with 106 μM CFZ (red lines) or DMSO (black lines) in medium containing either 2 μM iron (open circles), 190 μM iron (closed circles) or 950 μM iron (triangles). Cultures were plated for CFU at indicated timepoints. Data for panels **(A,B)** represent the mean ± SEM of four experimental replicates. Comparison between conditions for panel **(B)** was performed using unpaired student’s t-test. Analysis of panel **(B)** was performed using a Kruskal-Wallis test with lowest significance for all compared conditions represented. *p > 0.05; **p > 0.01; ***p > 0.001.

Given the apparent interaction between Fe^2+^ and CFZ, we next measured the impact of CFZ on the oxidation state of iron. Anaerobic *M. tuberculosis* culture conditions were used to prevent the rapid reoxidation of Fe^2+^ in the presence of oxygen. Reduced iron was assayed in the presence of 190 μM iron and a 5-fold excess (950 μM) of iron supplement using the Fe^2+^-specific chelator, ferene (Eskelinen et al., 1983). CFZ exposure led to a considerable increase in the rate of Fe^2+^ accumulation (Figure 8A). Accumulation of Fe^2+^ at a faster rate and to higher levels was observed at 950 μM iron compared to 190 μM iron; although, both CFZ-exposed conditions generated significantly more Fe^2+^ than controls.

We also investigated the role of iron availability on anaerobic CFZ activity. *M. tuberculosis* cultures grown in either 2 μM, 190 μM or 950 μM iron (5-times base iron supplement) for 8 days in an anaerobic *M. tuberculosis* dormancy model (Leistikow et al., 2010) were treated with 106 μM CFZ and plated for survival (Figure 8B). In a reversal of the role of iron concentration in aerobic conditions, higher levels of media iron displayed lower CFZ killing with an 8 day drop in CFU of 1.7-log_10_ at 950 μM iron, 2.1-log_10_ at 190 μM iron, and 2.7-log_10_ at 2 μM iron. DMSO control cultures were not reduced in viability over the 8 days of anaerobiosis and were not differentially affected by iron availability. These findings demonstrated that iron reduction correlated with decreased CFZ killing in anaerobic conditions and further indicate an interaction between CFZ activity and iron homeostasis.

## Discussion

In this study, we investigated the relationship between iron homeostatic mechanisms and the activity of CFZ against *M. smegmatis* and *M. tuberculosis*. A surprising finding was the ability of MBT to prevent CFZ lethality. The decreased killing activity of CFZ in low iron conditions relative to high iron conditions was reversed by deletion of the *mbtD* gene or chemical inhibition of mycobactin synthesis via administration of salicyl-AMS to inhibit MbtA. MbtA and MbtD catalyze unique reactions necessary for MBT production. Moreover, addition of exogenous MBTJ complemented *∆mbtD* and reversed the increased killing activity observed in both the *∆mbtD* strain and the salicyl-AMS-treated *M. smegmatis*. Therefore, the production of MBT by the bacilli is not necessary to prevent CFZ killing provided the bacilli have access to the siderophore, either produced by *M. smegmatis* or supplied exogenously. While the inhibition of MbtD or MbtA blocks the production of both MBT and cMBT (Wells et al., 2013; Lun et al., 2013), the fact that MBTJ alone rescued ∆*mbtD* and salicyl-AMS-treated *M. smegmatis* from CFZ killing demonstrates that cell-associated MBT is sufficient to inhibit CFZ without the need for cMBT. Absence of a second *M. smegmatis* siderophore, exochelin, did not increase CFZ lethality as observed with the loss of MBT, and even decreased CFZ lethality. Therefore, we conclude that the MBT/cMBT iron acquisition system is the sole mediator of this phenomenon while deemphasizing a protective role for general increased iron acquisition. Conditions which reduced the need for MBT made mycobacteria more susceptible to CFZ. These findings indicate the utilization of specific iron acquisition systems may provide an explanation for CFZ’s varied bactericidal activity across model systems and different mycobacterial species. For example, *M. leprae* lacks the capacity to produce and utilize MBT (Kato, 1985), possibly contributing to the pathogen’s considerable susceptibility to CFZ (Rees, 1969; Reddy et al., 1999).

Another interesting finding was that the presence of MBT was not sufficient to prevent killing but required conditions in which MBT was actively recycled. The observation that the addition of MBTJ did not protect *M. smegmatis* cultures grown in high iron medium demonstrates that the presence of MBT alone is not sufficient to block CFZ lethality under these conditions. *M. smegmatis* challenged with CFZ at high iron did not produce MBT and exogenous addition of MBTJ did not reduce lethality. It is likely under high iron conditions, MBT is not utilized by *M. smegmatis* even if present. *M. smegmatis* has several independent mechanisms for acquiring Fe^3+^ and can compensate for the lack of MBT under iron-limiting conditions (Siegrist et al., 2009), likely by preferentially using exochelin which typically serves as the main mechanism of *M. smegmatis* iron acquisition (Ratledge and Ewing, 1996). These additional mechanisms of iron acquisition likely contributed to the lack of MBT utilization even under CFZ challenge. Minimal MBT production by CFZ-treated *M. smegmatis* at low iron demonstrated that MBT was not the preferential mechanism to acquire additional iron during CFZ exposure as was observed in *M. tuberculosis*. Therefore, the transport system necessary for utilization and trafficking of MBT or MBTJ across the cell envelope may be absent under high iron conditions. Unlike *M. smegmatis*, *M. tuberculosis* is incapable of growth on free iron without access to MBT or cMBT; however, it does possess the capacity to access environmental heme with mechanisms largely independent of the MBT/cMBT system (Zhang et al., 2020; Wells et al., 2013). The co-utilization or preferred use of heme may explain why the protective effect of MBTJ was less substantial in ∆*mbtD M. tuberculosis* than observed in ∆*mbtD M. smegmatis*, as the latter would be primed to make full use of the MBTJ in the low iron environment.

The combination of our finding that MBT inhibits CFZ lethality and the numerous studies that demonstrate *mmpR5* mutants confer resistance to CFZ (Villellas et al., 2017; Ismail et al., 2019a; Hartkoorn et al., 2014), suggest a link between MBT transport and CFZ activity or efflux. MmpR5 negatively regulates production of the MBT MmpS5/L5 transporter (Hartkoorn et al., 2014; Alexander et al., 2017; Richard et al., 2019; Ismail et al., 2019b; Ismail et al., 2019a) in the presence of intracellular iron, which is also presumed to be a direct CFZ efflux pump. Increased utilization of the MmpS5/L5 efflux pump for MBT transport could compete for its ability to efflux CFZ which should increase CFZ activity. However, we observed loss of CFZ activity not increased CFZ activity under conditions in which MBT is present and presumably utilizing MmpS5/L5 for transport. It is likely that conditions in which mycobactins are required, such as low iron, induce higher MmpS5/L5 efflux pump expression. However, if the presence of mycobactins are required for use of the MmpS5/L5 pump, then the addition of MBTJ to a mycobactin mutant in low iron, but not high, could enable increased use of the MmpS5/L5 efflux pump shown to be important for CFZ activity (Meikle et al., 2023).

Interestingly, our findings demonstrate that the protective effect of MBT occurs only when present at the start of CFZ exposure. While concurrent addition of MBTJ and CFZ to ∆*mbtD M. smegmatis* rescued killing, addition of MBTJ addition 2 days after initial CFZ exposure increased killing. These findings suggest the increased killing observed when MBTJ is added 2 days after CFZ could be mediated by MBTJ competing with CFZ for efflux via the MmpS5/L5 transporter. However, competition between MBT and CFZ for efflux at the beginning of CFZ exposure does not appear to cause increased CFZ lethality as MBT prevents killing. Also supporting the temporal role for MBT utilization blocking CFZ is the observation that *M. tuberculosis* grown at high iron strongly induced *mbt* genes and produced detectable MBT within 24 h of CFZ exposure. However, these cultures that induced MBT only after CFZ addition died even more than cultures in low iron that were primed with considerable MBT prior to CFZ exposure. These findings demonstrate a link between CFZ activity and the mycobacterial utilization of MBT; however, the specific mechanism of this link requires further investigation to determine if it is solely due to changes in efflux activity or other interactions of CFZ with mycobactins and iron.

Another finding of this study concerns the relationship between CFZ and intracellular iron. The fact that iron-repressed genes, both IdeR-dependent and IdeR-independent, were upregulated in CFZ-treated *M. tuberculosis* indicates a disruption to normal iron homeostasis, a fact underscored by the production of substantial MBT by CFZ-exposed *M. tuberculosis* in high iron conditions. *M. smegmatis* challenged with CFZ at high iron did not increase MBT levels, an observation possibly explained by the multiple redundant mechanisms of acquiring environmental Fe^3+^ in *M. smegmatis* compared to *M. tuberculosis*, which is entirely dependent on MBT and cMBT (Wells et al., 2013). We also observed that CFZ induced intracellular ^55^Fe loss more strongly in *M. smegmatis* challenged with CFZ at low iron compared to high iron conditions. Iron loss was inversely correlated with CFZ lethality as CFZ is less lethal under low iron compared to high iron conditions. Therefore, if iron loss is the mechanism of CFZ lethality blocked by MBT, replacement of iron via MBT transport would need to overcome the increased iron efflux occurring in low relative to high iron conditions.

Previous work focused on the redox activity of CFZ demonstrated a role for the type-II NADH dehydrogenase (NDH-2) as it was observed that NDH-2 reduces CFZ within *M. smegmatis* membrane vesicles. NDH-2 was shown to donate electrons to CFZ_ox_ and that this activity led to increased oxidative stress (Yano et al., 2011). In another study, it was determined that deletion of *ndh2* in *M. tuberculosis* did not negatively impact the growth inhibitory activity of CFZ (Beites et al., 2019). However, it is possible CFZ may be reduced by other respiratory enzymes. Whether or not CFZ reduction by NDH-II is essential for killing, the phenomenon may provide a model by which the redox-active nature of CFZ enables MBT-bound iron to facilitate removal of the drug from the bacterial envelope. Given that both NDH-2 and MBT localize to the inner membrane, it is possible that CFZ_red_ may encounter oxidized Fe^3+^ bound to siderophore awaiting internalization by the IrtA/B system or encounter other sources of membrane-localized Fe^3+^. Were iron and reduced CFZ to interact, it is conceivable they would form a complex, like the one observed in our CFZ solubility studies, which could increase solubility in aqueous solutions more than oxidized hydrophobic CFZ. A decrease in the hydrophobic nature of CFZ may facilitate its egress from the bacterial membranes. The ability of membrane localized CFZ_red_ to reduce Fe^3+^ bound to MBT could account for the efflux of iron in aerobic conditions and the accumulation of extracellular Fe^2+^ observed under anaerobic conditions in which reduced iron is not readily re-oxidized. Thus, the redox interaction of iron bound to MBT with CFZ could impact iron transport and decrease the ability of CFZ to be maintained in the cell and explain why increased iron reduction correlated with decreased killing.

The inhibitory effect of MBT on CFZ lethality could involve other potential redox mechanisms. Recycling of MBT requires the reduction of the MBT-Fe^3+^ complex by IrtA/B to release Fe^2+^ from MBT (Arnold et al., 2020). It is possible that CFZ activity could involve reduction by IrtA/B in a manner analogous to Ndh2 CFZ reduction, as previously described (Yano et al., 2011). In this scenario, IrtA/B reduction of MBT bound iron could compete with CFZ reduction and limit potential oxidative stress generation mediated by reduced CFZ, while simultaneously explaining the low-iron gene expression observed in *M. tuberculosis* treated with CFZ by preventing the uptake of MBT-bound iron.

**Figure 9:**
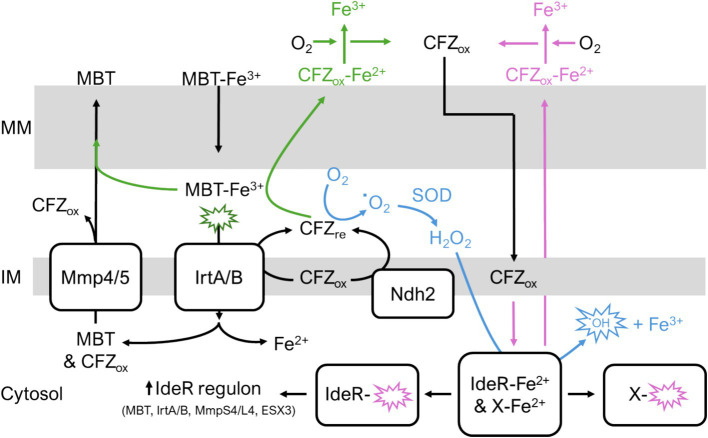
Potential interactions of CFZ with iron homeostasis. (Green pathway) CFZ becomes reduced by either Ndh2 or IrtA/B. Reduced CFZ reduces MBT-bound ferric iron. MBT releases ferrous iron allowing CFZ to bind the divalent cation which increases CFZ aqueous solubility and promotes CFZ egress from mycomembrane. Alternatively, MBT bound to ferric iron provides an alternative route to molecular oxygen for CFZ oxidation; thus, CFZ is maintained in an oxidized state unable to contribute to ROS in blue pathway. (Blue pathway) Reduced CFZ interacts with molecular oxygen resulting in superoxide generation and ROS that damages intracellular macromolecules including iron-containing proteins like IdeR and respiratory complexes. Demetallation of IdeR derepresses the IdeR regulon, inducing iron acquisition systems. (Pink pathway) CFZ directly chelates ferrous iron out of intracellular proteins. In these potential pathways, iron acquisition via MBT either rescues CFZ killing via replacement of intracellular iron or by providing an alternative mechanism of CFZ oxidation. MM, outer mycomembrane; IM, inner membrane.

Our study demonstrates a role for MBT in modulating CFZ activity. A limitation of this study is that additional experimentation is necessary to establish the precise mechanistic basis for MBT inhibition of CFZ lethality. One straightforward possibility is that CFZ requires direct interaction with iron for either solubility or activity. Thus, MBT inhibits CFZ via sequestration of iron. However, CFZ is known to interact with divalent cations and iron in aerated culture would be in the trivalent ferric form (Mohd et al., 2011). Also, the egress of cellular iron suggests iron would need to be replaced via iron acquisition systems (e.g., MBT, exochelin, and heme uptake). Figure 9 illustrates other potential mechanisms of MBT-mediated CFZ interference that are consistent with our findings. One possibility is that CFZ acts directly as an iron chelator as observed with pyrazolopyrimidinone which has antimycobacterial effects by targeting intracellular iron (Dragset et al., 2015). Ferric iron bound to MBT could also function as an alternate source for oxidation of CFZ reduced by Ndh2 or IrtA/B and therefore limit ROS generation. Lastly, ferric iron could be reduced by CFZ releasing ferrous iron to bind CFZ and promote CFZ aqueous solubility and egress from the lipophilic mycomembrane, preventing lethal quantities of CFZ from accumulating intracellularly.

Given our study and literature linking iron, MBT transport and CFZ resistance mechanisms, the presence of iron and MBT should be considered in future studies of CFZ activity against mycobacterial pathogens. Understanding the complex interactions of CFZ with mycobacterial iron acquisition systems may be crucial for understanding the efficacy of CFZ alone and in combination with other chemotherapeutic agents and for determining the optimal timing of CFZ administration. In particular, the impact of MBT utilization on CFZ may be important in assessing future combination therapies that contain bedaquiline as *mmpR5* mutants that confer CFZ resistance also confer resistance to bedaquiline. Moreover, *mmpR5* mutations are prevalent in nearly 11% of MDR-TB patients, a rate nearly 20 times higher than patients with drug-susceptible strains despite lack of CFZ or bedaquiline exposure (Villellas et al., 2017). As such, understanding this interaction will be critical in effectively deploying CFZ in accordance with new recommendations for the drug’s inclusion in MDR drug regimens, particularly alongside bedaquiline.

## Data Availability

The datasets presented in this study can be found in online repositories. The names of the repository/repositories and accession number(s) can be found at: https://www.ebi.ac.uk/arrayexpress/, E-MTAB-9350.

## Footnotes

^1^ www.ebi.ac.uk/arrayexpress
