# Supplementary material for: Mycobactin and clofazimine activity are negatively correlated in mycobacteria
Source: Front Microbiol. 2025 Apr 3;16:1539139. doi: 10.3389/fmicb.2025.1539139 (PMC12003420; doi:10.3389/fmicb.2025.1539139)
Supplement: Supplementary file 1 [file Table_1.docx]

**Supplemental Table 1: Genes significantly^a^ upregulated^b^ by CFZ exposure**

| **Erdman #** | | **Gene** |  | **Fold change** | | **P-value** |  | **Function^c^** |
| --- | --- | --- | --- | --- | --- | --- | --- | --- |
| 74 |  |  |  | 6.33 |  | 1.50E-02 |  | hypothetical protein |
| 125 |  |  |  | 2.28 |  | 8.66E-09 |  | hypothetical protein |
| 132 |  |  |  | 14.17 |  | 9.78E-03 |  | hypothetical protein |
| 137 |  | *oxcA* |  | 2.20 |  | 7.79E-03 |  | putative oxalyl-CoA decarboxylase |
| 155 |  |  |  | 2.04 |  | 1.58E-02 |  | acetyltransferase |
| 158 |  | *cyp138* |  | 3.83 |  | 2.75E-08 |  | cytochrome P450 138 |
| 168 |  |  |  | 2.39 |  | 1.43E-11 |  | hypothetical protein |
| 213 |  |  |  | 2.10 |  | 4.04E-04 |  | transmembrane protein |
| 215 |  |  |  | 3.32 |  | 2.37E-09 |  | CsoR family transcriptional regulator, copper-sensing transcriptional repressor |
| 218 |  |  |  | 8.03 |  | 0.00E+00 |  | hypothetical protein |
| 222 |  |  |  | 2.24 |  | 2.38E-02 |  | two component transcriptional regulatory protein |
| 235 |  | *trmB* |  | 2.34 |  | 1.89E-06 |  | tRNA (guanine-N(7))-methyltransferase |
| 236 |  |  |  | 2.29 |  | 4.57E-04 |  | hypothetical protein |
| 237 |  |  |  | 2.51 |  | 1.96E-02 |  | hypothetical protein |
| 238 |  | *pckA* |  | 2.46 |  | 2.43E-07 |  | phosphoenolpyruvate carboxykinase |
| 271 |  | *fabG_1* |  | 2.18 |  | 2.80E-07 |  | 3-ketoacyl-(acyl-carrier-protein) reductase |
| 272 |  | *fadA2* |  | 2.01 |  | 3.86E-05 |  | acetyl-CoA acetyltransferase |
| 273 |  | *fadE5* |  | 2.21 |  | 1.84E-09 |  | acyl-CoA dehydrogenase |
| 280 |  | *hsp* |  | 2.98 |  | 7.62E-03 |  | heat shock protein |
| 295 |  | *narU* |  | 2.15 |  | 2.76E-02 |  | integral membrane nitrite extrusion protein |
| 305 |  |  |  | 2.35 |  | 1.87E-09 |  | hypothetical protein |
| 316 |  |  |  | 3.80 |  | 0.00E+00 |  | hypothetical protein |
| 317 |  |  |  | 3.77 |  | 0.00E+00 |  | hypothetical protein |
| 318 |  |  |  | 3.33 |  | 0.00E+00 |  | DNA segregation ATPase FtsK/SpoIIIE, S-DNA-T family |
| 320 |  | *PPE4* |  | 3.17 |  | 0.00E+00 |  | PPE family protein |
| 321 |  | *esxG* |  | 4.08 |  | 3.36E-11 |  | hypothetical protein |
| 322 |  | *esxH* |  | 2.73 |  | 3.28E-13 |  | low molecular weight protein antigen 7 |
| 323 |  |  |  | 3.08 |  | 7.24E-14 |  | hypothetical protein |
| 324 |  |  |  | 2.98 |  | 1.94E-13 |  | transmembrane protein |
| 325 |  | *mycP3* |  | 3.23 |  | 5.40E-14 |  | membrane-anchored mycosin |
| 326 |  | *eccE3* |  | 2.25 |  | 1.22E-07 |  | ESX-3 secretion system protein |
| 331 |  |  |  | 3.94 |  | 1.13E-11 |  | PE-PGRS family protein |
| 332 |  |  |  | 3.51 |  | 1.53E-05 |  | PE-PGRS family protein |
| 339 |  | *PE5* |  | 3.96 |  | 0.00E+00 |  | PPE family protein |
| 457 |  | *thiO* |  | 2.54 |  | 2.06E-03 |  | thiamine biosynthesis oxidoreductase |
| 458 |  | *thiS* |  | 9.96 |  | 1.59E-03 |  | sulfur carrier protein |
| 459 |  | *thiG* |  | 2.95 |  | 2.50E-06 |  | thiazole synthase |
| 492 |  |  |  | 2.18 |  | 7.29E-04 |  | hypothetical protein |
| 493 |  | *mmpL4* |  | 5.25 |  | 0.00E+00 |  | transmembrane transport protein |
| 494 |  | *mmpS4* |  | 7.85 |  | 0.00E+00 |  | membrane protein |
| 496 |  |  |  | 124.88 |  | 1.30E-02 |  | hypothetical protein |
| 510 |  |  |  | 4.02 |  | 0.00E+00 |  | transcriptional regulator |
| 512 |  | *icl* |  | 12.28 |  | 0.00E+00 |  | isocitrate lyase |
| 513 |  | *fadB2* |  | 4.28 |  | 0.00E+00 |  | 3-hydroxybutyryl-CoA dehydrogenase |
| 514 |  | *umaA* |  | 3.39 |  | 9.99E-16 |  | mycolic acid synthase |
| 522 |  |  |  | 2.47 |  | 2.26E-05 |  | transmembrane protein |
| 531 |  |  |  | 2.55 |  | 1.84E-11 |  | transcriptional regulator |
| 539 |  |  |  | 5.68 |  | 1.21E-02 |  | hypothetical protein |
| 625 |  |  |  | 2.25 |  | 3.03E-02 |  | putative phosphoribosyl transferase |
| 631 |  |  |  | 14.46 |  | 3.89E-10 |  | hypothetical protein |
| 634 |  |  |  | 5.17 |  | 8.60E-04 |  | PE-PGRS family protein |
| 638 |  |  |  | 2.12 |  | 2.87E-04 |  | hypothetical protein |
| 647 |  | *mce2A* |  | 4.98 |  | 2.57E-03 |  | MCE-family protein |
| 718 |  | *fabD* |  | 4.14 |  | 0.00E+00 |  | malonyl CoA-acyl carrier protein transacylase |
| 744 |  | *echA4* |  | 4.48 |  | 1.28E-02 |  | enoyl-CoA hydratase |
| 747 |  | *mmpL5* |  | 2.02 |  | 1.23E-07 |  | transmembrane transport protein |
| 749 |  |  |  | 2.89 |  | 1.84E-07 |  | hypothetical protein |
| 751 |  |  |  | 2.17 |  | 1.03E-03 |  | transmembrane protein |
| 763 |  |  |  | 2.57 |  | 3.57E-04 |  | hypothetical protein |
| 764 |  |  |  | 2.02 |  | 4.86E-04 |  | hypothetical protein |
| 783 |  | *atsA* |  | 2.28 |  | 7.78E-08 |  | arylsulfatase |
| 825 |  |  |  | 6.04 |  | 9.70E-08 |  | PE-PGRS family protein |
| 836 |  |  |  | 2.41 |  | 8.61E-03 |  | transposase |
| 887 |  |  |  | 2.26 |  | 2.25E-02 |  | hypothetical protein |
| 906 |  |  |  | 8.70 |  | 0.00E+00 |  | transcriptional regulator |
| 907 |  | *desA1* |  | 5.94 |  | 0.00E+00 |  | acyl-[acyl-carrier protein] desaturase |
| 911 |  |  |  | 19.67 |  | 9.88E-03 |  | deaminase |
| 922 |  |  |  | 5.42 |  | 1.36E-11 |  | PE_PGRS family protein |
| 925 |  | *lpqQ* |  | 2.59 |  | 1.43E-03 |  | lipoprotein |
| 930 |  |  |  | 2.61 |  | 4.27E-05 |  | hypothetical protein |
| 939 |  | *lpqS* |  | 11.21 |  | 0.00E+00 |  | lipoprotein |
| 940 |  | *cysK2* |  | 5.03 |  | 1.26E-13 |  | cysteine synthase A |
| 941 |  |  |  | 4.16 |  | 0.00E+00 |  | integral membrane transport protein |
| 942 |  |  |  | 11.06 |  | 2.27E-10 |  | transposase |
| 943 |  |  |  | 2.32 |  | 2.97E-03 |  | short chain dehydrogenase |
| 964 |  |  |  | 3.06 |  | 1.98E-09 |  | PE-PGRS family protein |
| 967 |  |  |  | 2.31 |  | 1.81E-02 |  | hypothetical protein |
| 978 |  |  |  | 12.67 |  | 0.00E+00 |  | hypothetical protein |
| 979 |  | *fprB* |  | 19.53 |  | 0.00E+00 |  | NADPH:adrenodoxin oxidoreductase |
| 980 |  |  |  | 5.93 |  | 6.14E-04 |  | hypothetical protein |
| 1039 |  |  |  | 2.53 |  | 4.58E-07 |  | ATP-dependent DNA ligase |
| 1049 |  |  |  | 11.26 |  | 4.77E-02 |  | hypothetical protein |
| 1060 |  |  |  | 2.13 |  | 3.77E-04 |  | magnesium chelatase |
| 1061 |  |  |  | 2.07 |  | 2.96E-04 |  | hypothetical protein |
| 1091 |  | *mprA* |  | 2.76 |  | 1.57E-07 |  | two component response transcriptional regulatory protein |
| 1118 |  | *pabB* |  | 2.03 |  | 1.92E-02 |  | para-aminobenzoate synthase component I |
| 1135 |  |  |  | 2.49 |  | 5.90E-03 |  | TetR family transcriptional regulator |
| 1151 |  | *trcR* |  | 3.43 |  | 4.45E-07 |  | two component transcriptional regulator |
| 1165 |  |  |  | 13.11 |  | 1.16E-04 |  | hypothetical protein |
| 1179 |  |  |  | 11.73 |  | 0.00E+00 |  | hypothetical protein |
| 1187 |  |  |  | 5.92 |  | 1.01E-13 |  | hypothetical protein |
| 1188 |  |  |  | 7.22 |  | 3.21E-05 |  | hypothetical protein |
| 1189 |  |  |  | 6.70 |  | 4.34E-02 |  | PE-PGRS family protein |
| 1196 |  |  |  | 3.02 |  | 1.04E-03 |  | hypothetical protein |
| 1213 |  |  |  | 6.99 |  | 4.82E-03 |  | PE-PGRS family protein |
| 1226 |  | *desA2* |  | 3.53 |  | 1.11E-16 |  | acyl-[acyl-carrier protein] desaturase |
| 1237 |  |  |  | 2.64 |  | 7.18E-03 |  | carboxylesterase superfamily protein |
| 1276 |  |  |  | 4.00 |  | 1.97E-03 |  | hypothetical protein |
| 1289 |  |  |  | 4.60 |  | 6.97E-04 |  | transposase |
| 1302 |  | *phhB* |  | 2.56 |  | 1.09E-04 |  | pterin-4-alpha-carbinolamine dehydratase |
| 1311 |  | *PPE17* |  | 2.58 |  | 3.60E-09 |  | PPE family protein |
| 1312 |  | *PE11* |  | 2.54 |  | 1.57E-03 |  | PE family protein |
| 1320 |  | *fdxC* |  | 2.41 |  | 1.85E-09 |  | ferredoxin |
| 1322 |  |  |  | 2.19 |  | 2.52E-04 |  | hypothetical protein |
| 1323 |  | *pks4* |  | 4.68 |  | 0.00E+00 |  | polyketide beta-ketoacyl synthase |
| 1324 |  | *papA3* |  | 4.90 |  | 0.00E+00 |  | polyketide synthase associated protein |
| 1325 |  | *mmpL10* |  | 6.95 |  | 0.00E+00 |  | transmembrane transport protein |
| 1326 |  |  |  | 3.84 |  | 1.82E-10 |  | hypothetical protein |
| 1327 |  | *fadD21* |  | 3.90 |  | 0.00E+00 |  | acyl-CoA synthetase |
| 1329 |  | *rocA* |  | 6.63 |  | 2.68E-12 |  | pyrroline-5-carboxylate dehydrogenase |
| 1330 |  |  |  | 3.36 |  | 1.48E-02 |  | proline dehydrogenase |
| 1338 |  | *PE13* |  | 2.42 |  | 5.05E-03 |  | PE family protein |
| 1354 |  |  |  | 3.40 |  | 8.48E-04 |  | hypothetical protein |
| 1361 |  |  |  | 2.80 |  | 7.35E-05 |  | integral membrane protein |
| 1362 |  |  |  | 2.60 |  | 4.55E-05 |  | tetronasin-transport integral membrane proteinABC transporter |
| 1363 |  |  |  | 2.12 |  | 1.62E-04 |  | tetronasin-transport ATP-binding protein ABCtransporter |
| 1364 |  |  |  | 2.06 |  | 6.72E-05 |  | transcriptional regulator |
| 1393 |  |  |  | 2.11 |  | 2.22E-02 |  | hypothetical protein |
| 1473 |  | *ogt* |  | 3.81 |  | 4.04E-05 |  | methylated-DNA-protein-cysteine methyltransferase |
| 1501 |  |  |  | 2.84 |  | 6.18E-06 |  | putative deoxyribonucleotide triphosphatepyrophosphatase |
| 1502 |  |  |  | 14.52 |  | 0.00E+00 |  | hypothetical protein |
| 1503 |  | *lprD* |  | 22.11 |  | 0.00E+00 |  | lipoprotein |
| 1504 |  |  |  | 40.39 |  | 0.00E+00 |  | acyl carrier protein |
| 1505 |  | *fadD33* |  | 21.18 |  | 0.00E+00 |  | acyl-CoA synthetase |
| 1506 |  | *fadE14* |  | 30.18 |  | 0.00E+00 |  | acyl-CoA dehydrogenase |
| 1507 |  |  |  | 11.67 |  | 0.00E+00 |  | lysine N-acyltransferase |
| 1509 |  | *irtA* |  | 11.83 |  | 0.00E+00 |  | ATP-binding cassette, subfamily B |
| 1510 |  | *irtB* |  | 10.13 |  | 0.00E+00 |  | ATP-binding cassette, subfamily B |
| 1512 |  |  |  | 2.80 |  | 1.22E-05 |  | hypothetical protein |
| 1525 |  | *rsfA* |  | 12.06 |  | 1.01E-08 |  | anti-anti-sigma factor |
| 1534 |  |  |  | 11.55 |  | 0.00E+00 |  | ribosomal protein S12 methylthiotransferase accessory factor |
| 1535 |  |  |  | 4.49 |  | 0.00E+00 |  | hypothetical protein |
| 1545 |  | *PE20* |  | 231.66 |  | 5.71E-03 |  | PPE family protein |
| 1551 |  |  |  | 3.31 |  | 9.12E-07 |  | monoxygenase |
| 1560 |  | *priA* |  | 2.15 |  | 3.38E-02 |  | primosome assembly protein |
| 1561 |  |  |  | 3.27 |  | 5.22E-09 |  | putative methyltransferase |
| 1563 |  |  |  | 46.74 |  | 0.00E+00 |  | putative methyltransferase |
| 1571 |  | *ribC* |  | 2.29 |  | 2.68E-02 |  | riboflavin synthase subunit alpha |
| 1578 |  |  |  | 2.14 |  | 6.99E-07 |  | hypothetical protein |
| 1626 |  |  |  | 13.83 |  | 0.00E+00 |  | transcriptional regulator |
| 1627 |  | *sufB* |  | 10.91 |  | 0.00E+00 |  | Fe-S cluster assembly protein SufB |
| 1628 |  | *sufD* |  | 12.53 |  | 0.00E+00 |  | Fe-S cluster assembly protein SufD |
| 1629 |  |  |  | 9.21 |  | 0.00E+00 |  | Fe-S cluster assembly ATP-binding protein |
| 1630 |  | *csd* |  | 11.75 |  | 0.00E+00 |  | cysteine desulfurase |
| 1631 |  |  |  | 9.88 |  | 0.00E+00 |  | nitrogen fixation related protein |
| 1632 |  |  |  | 11.43 |  | 0.00E+00 |  | hypothetical protein |
| 1635 |  |  |  | 2.36 |  | 4.03E-04 |  | PE-PGRS family protein |
| 1657 |  |  |  | 2.18 |  | 2.32E-02 |  | hypothetical protein |
| 1692 |  |  |  | 6.42 |  | 8.35E-05 |  | hypothetical protein |
| 1693 |  |  |  | 28.54 |  | 6.23E-14 |  | hypothetical protein |
| 1694 |  |  |  | 2.10 |  | 4.48E-06 |  | sugar transferase |
| 1706 |  | *adh* |  | 2.14 |  | 3.09E-02 |  | alcohol dehydrogenase |
| 1711 |  |  |  | 2.40 |  | 1.01E-02 |  | hypothetical protein |
| 1733 |  | *mmpL6* |  | 6.17 |  | 0.00E+00 |  | transmembrane transport protein |
| 1752 |  |  |  | 4.32 |  | 0.00E+00 |  | hypothetical protein |
| 1782 |  | *cydB* |  | 2.20 |  | 4.09E-07 |  | integral membrane cytochrome D ubiquinoloxidase subunit II |
| 1803 |  |  |  | 2.54 |  | 4.13E-03 |  | hypothetical protein |
| 1804 |  |  |  | 2.34 |  | 7.76E-08 |  | hypothetical protein |
| 1812 |  |  |  | 3.20 |  | 2.15E-08 |  | hypothetical protein |
| 1813 |  |  |  | 2.80 |  | 5.16E-11 |  | transmembrane protein |
| 1847 |  | *moeX* |  | 3.93 |  | 2.12E-04 |  | molybdopterin biosynthesis protein |
| 1848 |  |  |  | 3.30 |  | 2.74E-06 |  | coiled-coil structural protein |
| 1857 |  | *lprJ* |  | 2.46 |  | 1.07E-05 |  | lipoprotein |
| 1873 |  | *PPE22* |  | 2.04 |  | 3.71E-03 |  | PPE family protein |
| 1937 |  |  |  | 4.47 |  | 4.73E-05 |  | molybdopterin oxidoreductase |
| 1939 |  | *cut1* |  | 3.41 |  | 3.16E-14 |  | cutinase |
| 1942 |  |  |  | 10.03 |  | 4.25E-02 |  | PE-PGRS family protein |
| 1962 |  |  |  | 2.48 |  | 8.40E-05 |  | transcriptional regulator |
| 1977 |  | *PE18* |  | 2.09 |  | 2.03E-02 |  | PE family protein |
| 1979 |  | *PPE27* |  | 5.64 |  | 2.08E-03 |  | PPE family protein |
| 1991 |  | *PPE29* |  | 10.19 |  | 3.99E-02 |  | PPE family protein |
| 1993 |  | *PE_PGRS32* |  | 2.05 |  | 3.07E-04 |  | PE-PGRS family protein |
| 1994 |  |  |  | 6.24 |  | 6.93E-03 |  | hypothetical protein |
| 1997 |  | *PPE32* |  | 2.77 |  | 4.49E-04 |  | PPE family protein |
| 1998 |  | *PPE33* |  | 2.61 |  | 7.84E-07 |  | PPE family protein |
| 2035 |  |  |  | 2.50 |  | 1.82E-06 |  | transcriptional regulator |
| 2053 |  | *adhA* |  | 2.12 |  | 3.65E-03 |  | alcohol dehydrogenase |
| 2102 |  | *furA* |  | 2.60 |  | 7.98E-03 |  | ferric uptake regulation protein A |
| 2110 |  | *PPE34* |  | 11.76 |  | 1.85E-08 |  | PPE family protein |
| 2146 |  |  |  | 4.02 |  | 1.05E-02 |  | hypothetical protein |
| 2152 |  |  |  | 2.52 |  | 1.14E-04 |  | hypothetical protein |
| 2169 |  | *lprM* |  | 101.98 |  | 1.69E-02 |  | MCE-family lipoprotein |
| 2185 |  |  |  | 2.65 |  | 8.62E-04 |  | hypothetical protein |
| 2230 |  |  |  | 2.15 |  | 8.26E-03 |  | hypothetical protein |
| 2243 |  | *hspX* |  | 18.59 |  | 3.38E-04 |  | heat shock protein |
| 2250 |  |  |  | 2.34 |  | 1.71E-02 |  | transmembrane protein |
| 2265 |  |  |  | 2.03 |  | 1.44E-02 |  | hypothetical protein |
| 2279 |  | *cobG* |  | 2.64 |  | 7.45E-03 |  | cobalamin biosynthesis protein |
| 2312 |  | *tatA* |  | 2.28 |  | 1.05E-07 |  | twin argininte translocase |
| 2338 |  | *hisE* |  | 6.41 |  | 3.22E-10 |  | phosphoribosyl-ATP pyrophosphatase |
| 2339 |  | *PPE37* |  | 58.47 |  | 0.00E+00 |  | PPE family protein |
| 2354 |  |  |  | 2.90 |  | 1.44E-12 |  | hypothetical protein |
| 2361 |  |  |  | 2.58 |  | 3.10E-02 |  | hypothetical protein |
| 2378 |  |  |  | 3.69 |  | 9.60E-10 |  | hypothetical protein |
| 2379 |  |  |  | 4.08 |  | 4.02E-07 |  | hypothetical protein |
| 2380 |  |  |  | 2.99 |  | 1.10E-11 |  | hypothetical protein |
| 2386 |  |  |  | 2.46 |  | 4.47E-09 |  | transmembrane protein |
| 2447 |  |  |  | 2.09 |  | 4.56E-02 |  | hypothetical protein |
| 2469 |  | *acpP* |  | 3.56 |  | 0.00E+00 |  | acyl carrier protein |
| 2470 |  | *kasA* |  | 3.65 |  | 0.00E+00 |  | 3-oxoacyl-(acyl carrier protein) synthase II |
| 2471 |  | *kasB* |  | 3.89 |  | 0.00E+00 |  | 3-oxoacyl-(acyl carrier protein) synthase II |
| 2472 |  | *accD6* |  | 4.93 |  | 0.00E+00 |  | acetyl/propionyl-CoA carboxylase beta subunit |
| 2473 |  |  |  | 4.41 |  | 0.00E+00 |  | hypothetical protein |
| 2480 |  |  |  | 2.15 |  | 4.00E-02 |  | Lrp family conserved hypothetical transcriptional regulator |
| 2552 |  | *narK1* |  | 2.19 |  | 4.80E-07 |  | nitrite extrusion protein 1 |
| 2553 |  | *lppP* |  | 2.85 |  | 7.19E-04 |  | lipoprotein |
| 2562 |  |  |  | 11.38 |  | 2.73E-08 |  | probable transposase |
| 2568 |  | *PE_PGRS39* | | 2.29 |  | 1.03E-02 |  | PE-PGRS family protein |
| 2591 |  |  |  | 2.24 |  | 8.65E-04 |  | ArsR family transcriptional regulator |
| 2592 |  | *furB* |  | 4.19 |  | 1.66E-08 |  | ferric uptake regulation protein B |
| 2612 |  | *mbtH* |  | 23.14 |  | 0.00E+00 |  | MbtH protein |
| 2613 |  | *mbtG* |  | 21.48 |  | 0.00E+00 |  | lysine-N-oxygenase |
| 2614 |  | *mbtF* |  | 15.53 |  | 0.00E+00 |  | mycobactin peptide synthetase MbtF |
| 2615 |  | *mbtE* |  | 26.05 |  | 0.00E+00 |  | mycobactin peptide synthetase MbtE |
| 2616 |  | *mbtD* |  | 34.13 |  | 0.00E+00 |  | mycobactin polyketide synthetase MbtD |
| 2617 |  | *mbtC* |  | 59.52 |  | 0.00E+00 |  | mycobactin polyketide synthetase MbtC |
| 2618 |  | *mbtB* |  | 44.14 |  | 0.00E+00 |  | mycobactin phenyloxazoline synthetase |
| 2619 |  | *mbtA* |  | 13.21 |  | 0.00E+00 |  | mycobactin salicyl-AMP ligase |
| 2620 |  | *mbtJ* |  | 42.10 |  | 0.00E+00 |  | putative acetyl hydrolase |
| 2621 |  | *mbtI* |  | 20.77 |  | 0.00E+00 |  | salicylate synthase |
| 2624 |  | *hemN* |  | 2.08 |  | 3.48E-03 |  | coproporphyrinogen III oxidase |
| 2625 |  | *rpfD* |  | 4.62 |  | 3.00E-15 |  | resuscitation-promoting factor |
| 2626 |  |  |  | 8.00 |  | 0.00E+00 |  | Mce-associated membrane protein |
| 2628 |  | *cysH* |  | 2.01 |  | 1.98E-05 |  | phosphoadenosine phosphosulfate reductase |
| 2629 |  |  |  | 2.41 |  | 2.29E-03 |  | hypothetical protein |
| 2632 |  |  |  | 5.37 |  | 2.34E-10 |  | hypothetical protein |
| 2634 |  | *cysA1* |  | 2.11 |  | 2.26E-04 |  | sulfate-transport integral membrane protein ABC transporter |
| 2635 |  | *cysW* |  | 2.58 |  | 2.29E-05 |  | sulfate-transport integral membrane protein ABC transporter |
| 2637 |  | *subI* |  | 2.06 |  | 2.00E-04 |  | sulfate-binding lipoprotein |
| 2640 |  |  |  | 59.33 |  | 4.68E-02 |  | hypothetical protein |
| 2643 |  |  |  | 2.08 |  | 2.78E-03 |  | hypothetical protein |
| 2656 |  | *comEA* |  | 15.65 |  | 1.90E-02 |  | competence protein ComEA |
| 2672 |  | *ahpC* |  | 3.85 |  | 0.00E+00 |  | alkyl hydroperoxide reductase subunit C |
| 2673 |  | *ahpD* |  | 2.38 |  | 1.02E-08 |  | alkyl hydroperoxide reductase subunit D |
| 2699 |  | *mobA* |  | 2.44 |  | 6.31E-05 |  | molybdopterin-guanine dinucleotide biosynthesisprotein A |
| 2729 |  |  |  | 2.09 |  | 1.14E-05 |  | diacylglycerol O-acyltransferase / wax synthase |
| 2735 |  |  |  | 13.38 |  | 8.98E-03 |  | PE_PGRS family protein |
| 2769 |  |  |  | 2.61 |  | 8.32E-03 |  | hypothetical protein |
| 2799 |  | *lppA* |  | 2.29 |  | 2.81E-06 |  | lipoprotein |
| 2805 |  |  |  | 3.15 |  | 2.31E-02 |  | hypothetical protein |
| 2815 |  |  |  | 2.31 |  | 6.17E-05 |  | hypothetical protein |
| 2816 |  |  |  | 2.92 |  | 3.74E-06 |  | hypothetical protein |
| 2849 |  | *fadD9* |  | 3.29 |  | 0.00E+00 |  | fatty-acid-CoA ligase |
| 2850 |  | *PE_PGRS44* | | 2.41 |  | 7.47E-05 |  | PE-PGRS family protein |
| 2876 |  |  |  | 4.85 |  | 3.04E-09 |  | hypothetical protein |
| 2877 |  |  |  | 7.70 |  | 0.00E+00 |  | hypothetical protein |
| 2878 |  |  |  | 4.26 |  | 1.27E-06 |  | hypothetical protein |
| 2879 |  |  |  | 2.14 |  | 2.56E-02 |  | hypothetical protein |
| 2880 |  |  |  | 2.82 |  | 2.17E-03 |  | transmembrane protein |
| 2881 |  |  |  | 5.46 |  | 8.12E-08 |  | transcriptional regulator |
| 2882 |  |  |  | 2.05 |  | 4.42E-02 |  | methyltransferase |
| 2886 |  |  |  | 4.42 |  | 2.84E-02 |  | hypothetical protein |
| 2892 |  |  |  | 2.64 |  | 1.79E-05 |  | hypothetical protein |
| 2893 |  |  |  | 3.02 |  | 5.55E-16 |  | hypothetical protein |
| 2899 |  |  |  | 3.71 |  | 1.80E-05 |  | small multidrug resistance family-3 protein |
| 2900 |  |  |  | 3.85 |  | 1.11E-16 |  | ArsR family transcriptional regulator |
| 2914 |  |  |  | 2.66 |  | 4.06E-02 |  | phiRv2 prophage protein |
| 2939 |  | *hemY* |  | 2.20 |  | 3.12E-02 |  | protoporphyrinogen oxidase |
| 2956 |  |  |  | 2.08 |  | 2.15E-03 |  | integral membrane alanine and leucine rich protein |
| 2957 |  |  |  | 2.44 |  | 8.02E-09 |  | hypothetical protein |
| 2967 |  |  |  | 2.10 |  | 1.06E-02 |  | hypothetical protein |
| 2972 |  | *sigB* |  | 2.33 |  | 4.90E-08 |  | RNA polymerase sigma factor |
| 2990 |  |  |  | 3.11 |  | 2.06E-11 |  | hypothetical protein |
| 2991 |  |  |  | 2.26 |  | 2.07E-02 |  | hypothetical protein |
| 3010 |  |  |  | 3.15 |  | 1.75E-08 |  | transcriptional regulator |
| 3084 |  |  |  | 2.19 |  | 1.35E-07 |  | CRISPR-associated protein Cas2 |
| 3085 |  |  |  | 2.30 |  | 2.22E-06 |  | CRISP-associated protein Cas1 |
| 3088 |  | *csm5_1* |  | 4.21 |  | 1.42E-02 |  | CRISPR-associated protein |
| 3093 |  |  |  | 5.04 |  | 6.27E-03 |  | CRISPR-associated protein Csm2 |
| 3096 |  |  |  | 5.81 |  | 3.68E-02 |  | hypothetical protein |
| 3106 |  | *ugpB* |  | 2.24 |  | 4.47E-02 |  | sn-glycerol-3-phosphate-binding lipoprotein |
| 3121 |  | *efpA* |  | 3.47 |  | 0.00E+00 |  | integral membrane efflux protein A |
| 3164 |  |  |  | 2.06 |  | 1.95E-02 |  | transposase |
| 3208 |  |  |  | 2.05 |  | 2.72E-04 |  | hypothetical protein |
| 3273 |  | *leuD* |  | 4.16 |  | 2.60E-06 |  | isopropylmalate isomerase small subunit |
| 3274 |  | *leuC* |  | 3.40 |  | 5.45E-10 |  | isopropylmalate isomerase large subunit |
| 3290 |  | *ilvC* |  | 2.64 |  | 2.95E-10 |  | ketol-acid reductoisomerase |
| 3291 |  | *ilvH* |  | 3.52 |  | 1.49E-08 |  | acetolactate synthase 3 regulatory subunit |
| 3292 |  | *ilvB1* |  | 2.60 |  | 2.32E-09 |  | acetolactate synthase 1 catalytic subunit |
| 3305 |  | *lpqA* |  | 4.45 |  | 3.75E-14 |  | lipoprotein |
| 3309 |  |  |  | 10.12 |  | 1.42E-03 |  | transposase |
| 3338 |  |  |  | 6.72 |  | 0.00E+00 |  | monooxygenase |
| 3346 |  |  |  | 2.13 |  | 2.02E-03 |  | short chain dehydrogenase |
| 3351 |  | *fadE22* |  | 2.01 |  | 3.25E-03 |  | acyl-CoA dehydrogenase |
| 3374 |  |  |  | 2.22 |  | 2.99E-04 |  | monooxygenase |
| 3376 |  |  |  | 2.45 |  | 1.79E-02 |  | short-chain type dehydrogenase/reductase |
| 3377 |  | *adhD* |  | 2.23 |  | 1.68E-04 |  | zinc-type alcohol dehydrogenase |
| 3378 |  |  |  | 2.97 |  | 1.65E-04 |  | hypothetical protein |
| 3379 |  |  |  | 2.27 |  | 4.98E-04 |  | hypothetical protein |
| 3380 |  | *fadD13* |  | 2.64 |  | 6.61E-07 |  | chain-fatty-acid-CoA ligase |
| 3421 |  |  |  | 2.63 |  | 4.12E-03 |  | SARP family transcriptional regulator, regulator of embCAB operon |
| 3439 |  | *fadE24* |  | 4.49 |  | 0.00E+00 |  | acyl-CoA dehydrogenase |
| 3440 |  | *fadE23* |  | 4.40 |  | 0.00E+00 |  | acyl-CoA dehydrogenase |
| 3442 |  |  |  | 2.05 |  | 6.81E-05 |  | hypothetical protein |
| 3461 |  |  |  | 7.89 |  | 0.00E+00 |  | TetR family transcriptional regulator |
| 3462 |  |  |  | 19.27 |  | 0.00E+00 |  | dioxygenase |
| 3463 |  |  |  | 7.99 |  | 2.17E-04 |  | integral membrane protein |
| 3505 |  |  |  | 2.04 |  | 6.32E-07 |  | ABC transporter ATP-binding protein |
| 3512 |  |  |  | 3.26 |  | 1.15E-03 |  | ATP-dependent DNA helicase |
| 3550 |  |  |  | 2.17 |  | 1.61E-04 |  | TrkA domain protein |
| 3564 |  | *rubB* |  | 2.56 |  | 2.68E-04 |  | rubredoxin |
| 3565 |  | *rubA* |  | 3.63 |  | 3.22E-05 |  | rubredoxin |
| 3566 |  | *alkB* |  | 4.95 |  | 0.00E+00 |  | transmembrane alkane 1-monooxygenase |
| 3585 |  |  |  | 10.46 |  | 1.11E-16 |  | hypothetical protein |
| 3586 |  | *ctpC* |  | 4.69 |  | 0.00E+00 |  | metal cation transporter P-type ATPase |
| 3587 |  |  |  | 2.25 |  | 1.34E-03 |  | integral membrane protein |
| 3630 |  |  |  | 5.38 |  | 1.30E-04 |  | secreted protein antigen |
| 3655 |  |  |  | 10.01 |  | 0.00E+00 |  | hypothetical protein |
| 3662 |  |  |  | 5.81 |  | 3.90E-02 |  | PE-PGRS family protein |
| 3676 |  |  |  | 5.05 |  | 3.33E-16 |  | hypothetical protein |
| 3702 |  | *idsB* |  | 2.29 |  | 8.01E-04 |  | polyprenyl synthetase |
| 3710 |  | *acrA1* |  | 2.33 |  | 3.81E-05 |  | short chain dehydrogenase |
| 3722 |  |  |  | 19.24 |  | 2.50E-02 |  | hypothetical protein |
| 3723 |  |  |  | 39.38 |  | 0.00E+00 |  | hypothetical protein |
| 3724 |  |  |  | 12.92 |  | 0.00E+00 |  | hypothetical protein |
| 3726 |  |  |  | 2.07 |  | 1.20E-02 |  | transcriptional regulator |
| 3733 |  |  |  | 2.42 |  | 2.93E-11 |  | hypothetical protein |
| 3751 |  |  |  | 2.00 |  | 1.75E-02 |  | hypothetical protein |
| 3765 |  |  |  | 6.46 |  | 2.02E-03 |  | transmembrane protein |
| 3816 |  | *PE31* |  | 2.15 |  | 2.39E-04 |  | PE family protein |
| 3823 |  | *cpsA* |  | 2.48 |  | 4.22E-11 |  | hypothetical protein |
| 3827 |  | *lipF* |  | 3.22 |  | 1.64E-08 |  | esterase |
| 3846 |  | *fadE27* |  | 2.68 |  | 7.97E-04 |  | acyl-CoA dehydrogenase |
| 3849 |  |  |  | 4.07 |  | 8.05E-12 |  | PE-PGRS family protein |
| 3850 |  |  |  | 3.95 |  | 1.31E-04 |  | PE-PGRS family protein |
| 3854 |  | *fadD19* |  | 2.45 |  | 4.77E-03 |  | acyl-CoA synthetase |
| 3874 |  |  |  | 5.68 |  | 3.81E-03 |  | short chain dehydrogenase |
| 3958 |  |  |  | 12.37 |  | 0.00E+00 |  | hypothetical protein |
| 3959 |  |  |  | 13.56 |  | 0.00E+00 |  | hypothetical protein |
| 3960 |  |  |  | 6.83 |  | 0.00E+00 |  | hypothetical protein |
| 3962 |  |  |  | 3.08 |  | 1.20E-05 |  | monooxygenase |
| 3977 |  |  |  | 2.02 |  | 2.32E-05 |  | hypothetical protein |
| 4000 |  |  |  | 3.68 |  | 2.44E-03 |  | hypothetical protein |
| 4009 |  |  |  | 4.08 |  | 2.63E-03 |  | hypothetical protein |
| 4023 |  |  |  | 2.99 |  | 1.64E-11 |  | hypothetical protein |
| 4036 |  | *cyp137* |  | 2.08 |  | 6.26E-05 |  | cytochrome P450 137 |
| 4037 |  |  |  | 2.24 |  | 3.92E-06 |  | hypothetical protein |
| 4058 |  |  |  | 2.71 |  | 3.41E-02 |  | hypothetical protein |
| 4092 |  |  |  | 2.50 |  | 6.46E-08 |  | hypothetical protein |
| 4097 |  | *PPE67* |  | 2.11 |  | 4.46E-02 |  | PPE family protein |
| 4101 |  |  |  | 2.32 |  | 2.05E-04 |  | oxidoreductase |
| 4124 |  | *lpqH* |  | 2.30 |  | 2.15E-05 |  | 19 kDa lipoprotein antigen precursor |
| 4128 |  |  |  | 2.57 |  | 1.87E-11 |  | hypothetical protein |
| 4189 |  |  |  | 2.09 |  | 1.11E-06 |  | hypothetical protein |
| 4190 |  | *mmpL8* |  | 3.71 |  | 0.00E+00 |  | integral membrane transport protein |
| 4191 |  | *papA1* |  | 6.67 |  | 0.00E+00 |  | polyketide synthase associated protein |
| 4192 |  | *pks2* |  | 5.54 |  | 0.00E+00 |  | polyketide synthase |
| 4197 |  |  |  | 7.25 |  | 5.99E-06 |  | transcriptional regulator |
| 4205 |  | *pheA* |  | 2.58 |  | 1.65E-06 |  | prephenate dehydratase |
| 4206 |  |  |  | 88.79 |  | 0.00E+00 |  | hypothetical protein |
| 4207 |  |  |  | 11.47 |  | 1.53E-12 |  | transcriptional regulator |
| 4228 |  | *ethR* |  | 2.07 |  | 3.85E-03 |  | transcriptional regulatory repressor protein |
| 4235 |  |  |  | 4.19 |  | 7.24E-13 |  | hypothetical protein |

**^a^** p < 0.05

^b^ Expression increased 2-fold or more during CFZ exposure

^c^ Gene function determined using the Mycobrowser platform:

Kapopoulou A, Lew JM, Cole ST. The MycoBrowser portal: a comprehensive and manually annotated resource for mycobacterial genomes. Tuberculosis (Edinb). Jan 91(1):8-13 (2011)
