# Supplementary material for: Mycobactin and clofazimine activity are negatively correlated in mycobacteria
Source: Front Microbiol. 2025 Apr 3;16:1539139. doi: 10.3389/fmicb.2025.1539139 (PMC12003420; doi:10.3389/fmicb.2025.1539139)
Supplement: Supplementary file 2 [file Table_2.docx]

**Supplemental Table 2: Genes significantly^a^ downregulated^b^ by CFZ exposure**

| **Erdman #** | | **Gene** |  | **Fold change** | | **P-value** |  | **Function^c^** |
| --- | --- | --- | --- | --- | --- | --- | --- | --- |
| 13 |  | *ppiA* |  | -2.36 |  | 6.46E-10 |  | iron-regulated peptidyl-prolyl cis-transisomerase A |
| 62 |  | *rpsF* |  | -2.32 |  | 2.65E-04 |  | 30S ribosomal protein S6 |
| 72 |  | *celA1* |  | -3.14 |  | 1.68E-10 |  | endo-1,4-beta-glucanase |
| 115 |  |  |  | -7.54 |  | 1.03E-02 |  | hypothetical protein |
| 123 |  |  |  | -3.21 |  | 5.21E-04 |  | hypothetical protein |
| 152 |  |  |  | -2.32 |  | 2.92E-02 |  | hypothetical protein |
| 161 |  |  |  | -2.22 |  | 5.38E-04 |  | oxidoreductase |
| 190 |  |  |  | -2.37 |  | 1.31E-04 |  | GntR family transcriptional regulator |
| 193 |  | *yrbE1B* |  | -2.45 |  | 1.06E-09 |  | integral membrane protein |
| 194 |  | *mce1A* |  | -2.47 |  | 9.00E-11 |  | MCE-family protein |
| 195 |  | *mce1B* |  | -2.90 |  | 6.44E-15 |  | MCE-family protein |
| 196 |  | *mce1C* |  | -2.60 |  | 2.78E-12 |  | MCE-family protein |
| 197 |  | *mce1D* |  | -2.65 |  | 3.24E-12 |  | MCE-family protein |
| 198 |  | *lprK* |  | -2.37 |  | 8.52E-09 |  | MCE-family lipoprotein |
| 199 |  | *mce1F* |  | -2.43 |  | 6.81E-11 |  | MCE-family protein |
| 241 |  | *fadD4* |  | -2.10 |  | 1.24E-02 |  | acyl-CoA synthetase |
| 263 |  |  |  | -2.17 |  | 9.77E-04 |  | transmembrane protein |
| 266 |  | *lpqI* |  | -3.34 |  | 2.34E-06 |  | lipoprotein |
| 279 |  |  |  | -3.00 |  | 2.64E-04 |  | hypothetical protein |
| 314 |  | *PPE3* |  | -3.31 |  | 6.25E-07 |  | PPE family protein |
| 344 |  |  |  | -2.04 |  | 2.82E-02 |  | hypothetical protein |
| 349 |  |  |  | -2.18 |  | 5.03E-08 |  | beta-1,3-glucanase precursor |
| 350 |  |  |  | -2.04 |  | 2.49E-02 |  | muconolactone isomerase |
| 357 |  | *udgA* |  | -2.57 |  | 1.15E-02 |  | UDP-glucose 6-dehydrogenase |
| 374 |  |  |  | -4.68 |  | 0.00E-00 |  | iron-sulfur-binding reductase |
| 381 |  |  |  | -2.98 |  | 4.97E-06 |  | hypothetical protein |
| 461 |  | *lpqL* |  | -2.03 |  | 7.75E-07 |  | lipoprotein aminopeptidase |
| 472 |  | *def* |  | -2.00 |  | 2.08E-03 |  | peptide deformylase |
| 482 |  |  |  | -2.48 |  | 1.84E-06 |  | short chain dehydrogenase |
| 491 |  |  |  | -3.21 |  | 3.02E-05 |  | hypothetical protein |
| 505 |  |  |  | -2.03 |  | 1.67E-04 |  | hypothetical protein |
| 547 |  | *proC* |  | -2.47 |  | 1.19E-05 |  | pyrroline-5-carboxylate reductase |
| 568 |  |  |  | -2.45 |  | 1.93E-03 |  | hypothetical protein |
| 570 |  |  |  | -2.58 |  | 7.23E-04 |  | hypothetical protein |
| 588 |  | *galE3* |  | -2.69 |  | 3.32E-02 |  | UDP-glucose 4-epimerase |
| 598 |  |  |  | -2.04 |  | 2.29E-03 |  | hypothetical protein |
| 619 |  |  |  | -2.52 |  | 8.19E-03 |  | nucleotide-binding protein |
| 667 |  |  |  | -3.29 |  | 4.13E-02 |  | hypothetical protein |
| 674 |  |  |  | -11.93 |  | 2.04E-02 |  | hypothetical protein |
| 681 |  | *galTa* |  | -2.13 |  | 4.66E-02 |  | galactose-1-phosphate uridylyltransferase |
| 694 |  | *recB* |  | -2.41 |  | 5.11E-04 |  | exonuclease V beta chain |
| 740 |  |  |  | -2.24 |  | 4.49E-06 |  | hydrolase |
| 782 |  | *rpsQ* |  | -2.25 |  | 2.90E-04 |  | 30S ribosomal protein S17 |
| 793 |  | *rplR* |  | -2.16 |  | 2.14E-02 |  | 50S ribosomal protein L18 |
| 864 |  |  |  | -10.65 |  | 1.61E-02 |  | hypothetical protein |
| 976 |  |  |  | -2.13 |  | 2.13E-04 |  | hypothetical protein |
| 985 |  |  |  | -2.06 |  | 1.59E-03 |  | LuxR family transcriptional regulator |
| 986 |  |  |  | -2.35 |  | 1.08E-05 |  | transcriptional regulator |
| 987 |  |  |  | -2.21 |  | 2.20E-04 |  | monooxygenase |
| 990 |  |  |  | -4.85 |  | 2.29E-03 |  | hypothetical protein |
| 1021 |  |  |  | -2.28 |  | 8.19E-04 |  | transposase |
| 1025 |  | *mntH* |  | -2.56 |  | 2.70E-04 |  | manganese transport protein MntH |
| 1026 |  |  |  | -2.01 |  | 4.27E-04 |  | hypothetical protein |
| 1052 |  |  |  | -5.48 |  | 0.00E-00 |  | hypothetical protein |
| 1089 |  |  |  | -2.37 |  | 7.38E-03 |  | PE_PGRS family protein |
| 1129 |  | *lpqT* |  | -2.03 |  | 5.79E-03 |  | lipoprotein |
| 1167 |  |  |  | -6.80 |  | 3.96E-03 |  | IS1081, transposase |
| 1230 |  | *fumC* |  | -2.03 |  | 2.20E-05 |  | fumarate hydratase |
| 1265 |  |  |  | -4.36 |  | 1.36E-05 |  | transcriptional regulator |
| 1266 |  |  |  | -2.37 |  | 4.92E-09 |  | hypothetical protein |
| 1267 |  | *gltA1* |  | -2.08 |  | 4.03E-04 |  | methylcitrate synthase |
| 1281 |  |  |  | -2.02 |  | 2.69E-02 |  | short-chain type dehydrogenase/reductase |
| 1298 |  |  |  | -13.33 |  | 1.88E-02 |  | hypothetical protein |
| 1300 |  |  |  | -2.39 |  | 2.62E-05 |  | hypothetical protein |
| 1365 |  |  |  | -2.31 |  | 6.57E-03 |  | methyltransferase |
| 1379 |  |  |  | -2.04 |  | 3.34E-02 |  | hypothetical protein |
| 1397 |  | *lprE* |  | -2.49 |  | 1.29E-03 |  | lipoprotein |
| 1521 |  | *PPE19* |  | -2.21 |  | 9.36E-03 |  | PPE family protein |
| 1537 |  | *pyrR* |  | -2.68 |  | 2.16E-06 |  | pyrimidine regulatory protein |
| 1540 |  |  |  | -2.37 |  | 2.56E-02 |  | export or membrane protein |
| 1592 |  |  |  | -2.09 |  | 4.21E-05 |  | hypothetical protein |
| 1651 |  | *fabG1* |  | -2.34 |  | 2.11E-09 |  | 3-oxoacyl-[acyl-carrier protein] reductase |
| 1675 |  |  |  | -4.80 |  | 4.29E-02 |  | hypothetical protein |
| 1700 |  |  |  | -2.35 |  | 2.36E-03 |  | glycosyltransferase |
| 1721 |  |  |  | -2.32 |  | 9.90E-03 |  | hypothetical protein |
| 1750 |  | *bioB* |  | -2.18 |  | 2.00E-06 |  | biotin synthase |
| 1864 |  |  |  | -3.33 |  | 0.00E-00 |  | hypothetical protein |
| 1865 |  |  |  | -2.79 |  | 8.17E-07 |  | hypothetical protein |
| 1891 |  |  |  | -5.40 |  | 1.55E-02 |  | hypothetical protein |
| 1897 |  |  |  | -80.34 |  | 1.76E-11 |  | probable transposase |
| 2005 |  |  |  | -2.13 |  | 1.57E-05 |  | hypothetical protein |
| 2047 |  | *modB* |  | -2.84 |  | 2.28E-02 |  | molbdenum-transport integral membrane proteinABC transporter |
| 2056 |  |  |  | -2.33 |  | 1.96E-02 |  | short chain dehydrogenase |
| 2068 |  | *bfrA* |  | -3.51 |  | 1.22E-13 |  | bacterioferritin |
| 2076 |  | *rpfC* |  | -2.40 |  | 4.45E-08 |  | resuscitation-promoting factor |
| 2078 |  | *fbpB* |  | -5.53 |  | 0.00E-00 |  | secreted antigen 85-B |
| 2130 |  | *fadE18* |  | -2.89 |  | 2.07E-02 |  | acyl-CoA dehydrogenase |
| 2155 |  |  |  | -2.04 |  | 1.18E-02 |  | hypothetical protein |
| 2159 |  |  |  | -3.81 |  | 3.23E-02 |  | hypothetical protein |
| 2240 |  |  |  | -3.04 |  | 2.31E-02 |  | hypothetical protein |
| 2242 |  |  |  | -5.43 |  | 1.45E-04 |  | hypothetical protein |
| 2244 |  | *acg* |  | -6.87 |  | 7.35E-08 |  | hypothetical protein |
| 2246 |  |  |  | -2.16 |  | 1.65E-02 |  | hypothetical protein |
| 2290 |  |  |  | -2.32 |  | 1.13E-03 |  | hypothetical protein |
| 2356 |  |  |  | -2.03 |  | 4.33E-02 |  | hypothetical protein |
| 2366 |  |  |  | -2.98 |  | 4.03E-14 |  | hypothetical protein |
| 2407 |  |  |  | -3.92 |  | 1.82E-07 |  | NLP/P60 family protein |
| 2408 |  |  |  | -3.81 |  | 0.00E-00 |  | NLP/P60 family protein |
| 2450 |  |  |  | -2.23 |  | 5.19E-03 |  | hypothetical protein |
| 2492 |  |  |  | -2.51 |  | 1.87E-03 |  | putative cytochrome P |
| 2543 |  | *rocD2* |  | -7.44 |  | 2.29E-02 |  | ornithine aminotransferase |
| 2579 |  | *plcC* |  | -2.25 |  | 1.48E-05 |  | phospholipase C |
| 2580 |  | *plcB* |  | -2.34 |  | 2.98E-09 |  | membrane-associated phospholipase C |
| 2581 |  | *plcA* |  | -2.07 |  | 1.60E-05 |  | membrane-associated phospholipase C |
| 2584 |  | *PPE38* |  | -117.22 |  | 1.74E-02 |  | PPE family protein |
| 2586 |  |  |  | -31.61 |  | 5.00E-04 |  | probable transposase |
| 2714 |  |  |  | -2.50 |  | 8.39E-03 |  | hypothetical protein |
| 2765 |  |  |  | -2.12 |  | 4.20E-02 |  | hypothetical protein |
| 2818 |  |  |  | -2.07 |  | 4.33E-05 |  | hypothetical protein |
| 2884 |  |  |  | -4.28 |  | 1.64E-02 |  | hypothetical protein |
| 2885 |  |  |  | -3.48 |  | 1.82E-03 |  | hypothetical protein |
| 2887 |  |  |  | -7.37 |  | 1.55E-04 |  | hypothetical protein |
| 2910 |  |  |  | -3.53 |  | 1.26E-02 |  | hypothetical protein |
| 2935 |  |  |  | -2.57 |  | 9.19E-09 |  | integral membrane protein |
| 3013 |  | *ftsK* |  | -2.06 |  | 3.37E-06 |  | cell division transmembrane protein |
| 3118 |  |  |  | -2.85 |  | 2.69E-03 |  | hypothetical protein |
| 3140 |  |  |  | -12.16 |  | 4.18E-02 |  | hypothetical protein |
| 3141 |  |  |  | -3.25 |  | 1.28E-07 |  | penicillin-binding lipoprotein |
| 3188 |  | *rimM* |  | -2.17 |  | 2.02E-02 |  | 16S rRNA-processing protein |
| 3190 |  | *rpsP* |  | -2.75 |  | 7.65E-06 |  | 30S ribosomal protein S16 |
| 3192 |  | *dacB2* |  | -2.19 |  | 2.50E-02 |  | D-alanyl-D-alanine carboxypeptidase |
| 3198 |  |  |  | -2.73 |  | 1.30E-04 |  | hypothetical protein |
| 3209 |  |  |  | -2.01 |  | 5.82E-06 |  | hypothetical protein |
| 3218 |  | *drrA* |  | -2.68 |  | 9.95E-06 |  | ABC-2 type transport system ATP-binding protein |
| 3227 |  |  |  | -2.86 |  | 5.99E-03 |  | IS1533 transposase |
| 3243 |  |  |  | -3.46 |  | 4.00E-15 |  | methyltransferase |
| 3265 |  |  |  | -2.23 |  | 1.96E-02 |  | resolvase |
| 3276 |  |  |  | -3.12 |  | 1.39E-09 |  | hypothetical protein |
| 3287 |  |  |  | -24.58 |  | 1.35E-02 |  | hypothetical protein |
| 3301 |  | *gatC* |  | -3.03 |  | 7.44E-04 |  | aspartyl/glutamyl-tRNA amidotransferase subunit C |
| 3368 |  |  |  | -2.03 |  | 1.58E-02 |  | hydrolase |
| 3382 |  |  |  | -3.96 |  | 4.83E-04 |  | hypothetical protein |
| 3402 |  | *fprA* |  | -2.35 |  | 2.45E-04 |  | NADPH:adrenodoxin oxidoreductase |
| 3418 |  |  |  | -2.09 |  | 1.32E-02 |  | hypothetical protein |
| 3423 |  |  |  | -2.36 |  | 3.28E-03 |  | PPE family protein |
| 3431 |  |  |  | -403.62 |  | 1.54E-09 |  | hypothetical protein |
| 3445 |  | *nuoA* |  | -4.69 |  | 6.33E-15 |  | NADH dehydrogenase subunit A |
| 3446 |  | *nuoB* |  | -3.92 |  | 1.36E-11 |  | NADH dehydrogenase subunit B |
| 3447 |  | *nuoC* |  | -4.78 |  | 0.00E-00 |  | NADH dehydrogenase subunit C |
| 3448 |  | *nuoD* |  | -4.19 |  | 0.00E-00 |  | NADH dehydrogenase subunit D |
| 3449 |  | *nuoE* |  | -6.28 |  | 2.24E-12 |  | NADH dehydrogenase subunit E |
| 3450 |  |  |  | -5.86 |  | 1.31E-08 |  | NADH dehydrogenase subunit F |
| 3451 |  | *nuoF* |  | -3.14 |  | 1.24E-07 |  | NADH dehydrogenase subunit F |
| 3452 |  | *nuoG* |  | -4.25 |  | 0.00E-00 |  | NADH dehydrogenase subunit G |
| 3453 |  | *nuoH* |  | -2.71 |  | 1.25E-13 |  | NADH dehydrogenase subunit H |
| 3454 |  | *nuoI* |  | -2.10 |  | 3.56E-05 |  | NADH dehydrogenase subunit I |
| 3455 |  | *nuoJ* |  | -2.85 |  | 2.80E-07 |  | NADH dehydrogenase subunit J |
| 3456 |  | *nuoK* |  | -2.57 |  | 5.69E-03 |  | NADH dehydrogenase subunit K |
| 3457 |  | *nuoL* |  | -3.55 |  | 0.00E-00 |  | NADH dehydrogenase subunit L |
| 3458 |  | *nuoM* |  | -3.29 |  | 2.04E-14 |  | NADH dehydrogenase subunit M |
| 3459 |  | *nuoN* |  | -2.83 |  | 3.25E-12 |  | NADH dehydrogenase subunit N |
| 3521 |  |  |  | -2.04 |  | 2.46E-02 |  | hypothetical protein |
| 3581 |  | *wbbL1* |  | -2.02 |  | 2.58E-04 |  | dTDP-RHA:A-D-GlcNAc-diphosphoryl polyprenolA-3-L-rhamnosyl transferase |
| 3591 |  | *purE* |  | -3.20 |  | 6.30E-04 |  | 5-(carboxyamino)imidazole ribonucleotide mutase |
| 3609 |  | *pcd* |  | -2.45 |  | 6.81E-04 |  | piperideine-6-carboxilic acid dehydrogenase |
| 3628 |  |  |  | -2.01 |  | 3.44E-02 |  | hypothetical protein |
| 3638 |  |  |  | -2.14 |  | 3.08E-03 |  | hypothetical protein |
| 3647 |  | *dacB1* |  | -2.22 |  | 7.49E-05 |  | penicillin-binding protein |
| 3706 |  |  |  | -2.46 |  | 2.90E-02 |  | transposase |
| 3742 |  |  |  | -2.64 |  | 7.13E-03 |  | hypothetical protein |
| 3743 |  |  |  | -2.31 |  | 2.23E-02 |  | tRNA threonylcarbamoyladenosine biosynthesis protein TsaE |
| 3749 |  |  |  | -2.31 |  | 6.32E-04 |  | transposase |
| 3771 |  | *rplM* |  | -2.20 |  | 3.40E-03 |  | 50S ribosomal protein L13 |
| 3773 |  | *esxU* |  | -14.09 |  | 2.89E-02 |  | ESAT-6 like protein |
| 3810 |  | *mhpE* |  | -2.83 |  | 4.52E-02 |  | 4-hydroxy-2-ketovalerate aldolase |
| 3900 |  |  |  | -2.51 |  | 3.32E-02 |  | hypothetical protein |
| 3907 |  | *fadE31* |  | -2.31 |  | 1.38E-02 |  | acyl-CoA dehydrogenase |
| 3922 |  |  |  | -2.21 |  | 1.68E-03 |  | transcriptional regulator |
| 3937 |  | *mutY* |  | -3.12 |  | 8.26E-04 |  | adenine glycosylase |
| 3946 |  | *lysS2* |  | -2.09 |  | 3.87E-07 |  | lysyl-tRNA synthetase, class II |
| 3967 |  | *lpqG* |  | -3.37 |  | 2.81E-05 |  | lipoprotein |
| 3996 |  | *PE33* |  | -22.66 |  | 1.62E-02 |  | PE family protein |
| 4026 |  |  |  | -2.22 |  | 3.22E-02 |  | hypothetical protein |
| 4065 |  | *dnaQ* |  | -2.02 |  | 3.76E-04 |  | DNA polymerase III subunit |
| 4066 |  |  |  | -2.23 |  | 1.82E-02 |  | probable ligase |
| 4177 |  |  |  | -2.30 |  | 2.06E-08 |  | hypothetical protein |
| 4208 |  | *bfrB* |  | -4.53 |  | 5.82E-13 |  | bacterioferritin |
| 4217 |  |  |  | -4.39 |  | 7.25E-03 |  | hypothetical protein |

^a^ p < 0.05

^b^ Expression increased 2-fold or more during CFZ exposure

^c^ Gene function determined using the Mycobrowser platform:

Kapopoulou A, Lew JM, Cole ST. The MycoBrowser portal: a comprehensive and manually annotated resource for mycobacterial genomes. Tuberculosis (Edinb). Jan 91(1):8-13 (2011)
