# Supplementary material for: Mycobactin and clofazimine activity are negatively correlated in mycobacteria
Source: Front Microbiol. 2025 Apr 3;16:1539139. doi: 10.3389/fmicb.2025.1539139 (PMC12003420; doi:10.3389/fmicb.2025.1539139)
Supplement: Supplementary file 3 [file Data_Sheet_1.docx]

**Supplemental Figure Legends**

**
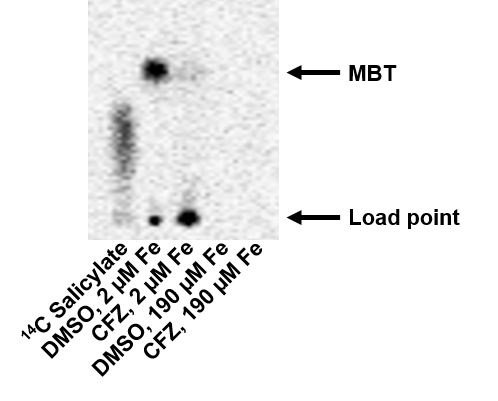
**

**Supplemental Figure 1. Exposure to CFZ did not induce MBT production in *M. smegmatis*.** Production of MBT by wild-type *M. smegmatis* cultures grown in low and high iron medium was visualized using ^14^C-salicylate, as previously described, after 24 hours of exposure to DMSO or CFZ.

**
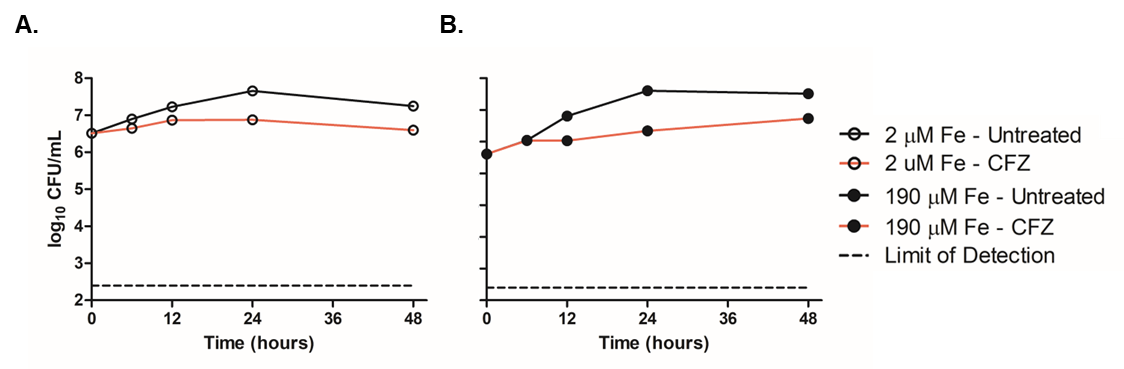
**

**Supplemental Figure 2. Iron efflux sampling was conducted prior to CFZ killing.** *M. smegmatis* cultures sampled for supernatant ^55^Fe in either (A) low or (B) high iron medium and treated with either 50 µM CFZ (red lines) or DMSO vehicle control (black lines) were plated for CFU at indicated timepoints.
